# Evaluation of Association Studies and an Updated Meta-Analysis of VDR Polymorphisms in Osteoporotic Fracture Risk

**DOI:** 10.3389/fgene.2021.791368

**Published:** 2022-01-07

**Authors:** Yi-yang Mu, Biao Liu, Bin Chen, Wang-fa Zhu, Xiang-Hua Ye, Hong-zhuo Li, Xiao-feng He

**Affiliations:** ^1^ Second Affiliated Hospital of Soochow University, Suzhou, China; ^2^ Department of Radiotherapy, First Affiliated Hospital, Zhejiang University School of Medicine, Hangzhou, China; ^3^ Department of Orthopaedics, Heping Hospital Affiliated to Changzhi Medical College, Changzhi, China; ^4^ Southern Medical University, Guangzhou, China

**Keywords:** VDR, polymorphism, osteoporosis, risk of fracture, meta-analysis

## Abstract

**Background:** Several studies have examined the association between vitamin D receptor (VDR) polymorphisms and osteoporotic fracture risk; however, the results are not uniform. Furthermore, many new articles have been published, and therefore, an updated meta-analysis was performed to further explore these issues.

**Objectives:** The aim of the study was to investigate the association between VDR, BsmI, ApaI, TaqI, FokI, and Cdx2 polymorphisms and osteoporotic fracture risk.

**Methods:** The odds ratios (ORs) and 95% confidence intervals (CIs) were used to assess the association between VDR BsmI, ApaI, TaqI, FokI, and Cdx2 polymorphisms and the risk of osteoporotic fracture. We also used the false-positive reporting probability (FPRP) test and the Venice criteria to evaluate the credibility of the statistically significant associations.

**Results:** Overall, this study found that the VDR ApaI and BsmI polymorphisms significantly increased the risk of osteoporotic fracture in European countries and America, respectively. However, when sensitivity analysis was performed after excluding low-quality and Hardy–Weinberg disequilibrium (HWD) studies, it was found that only individuals with the double-mutated genotype have an increased risk of osteoporotic fracture in European countries. In addition, when the credibility of the positive results was assessed, it was found that the positive results were not credible.

**Conclusion:** This meta-analysis indicates that there may be no significant association among the polymorphisms of VDR BsmI, ApaI, TaqI, FokI, and Cdx2 and the risk of osteoporotic fracture. The increased risk of osteoporotic fracture is most likely due to false-positive results.

## Introduction

Osteoporosis is characterized by reduced bone density and increased bone fragility, leading to an increased risk of fracture (Recker, 2005). Its clinical significance lies in the triggering of osteoporotic fractures (e.g., fractures of the forearm, vertebrae, and hip) (Cummings and Melton, 2002). The World Health Organization estimates that 200 million people worldwide suffer from osteoporosis (Uzzan et al., 2007), placing a huge burden on families and society, and that osteoporosis has become a major public health problem. Therefore, it is important to explore the underlying pathogenic factors.

The main factors in the development of osteoporosis encompass both environmental and genetic factors. The environmental factors include smoking, exercise, and alcohol consumption (Ng et al., 2006; Kaufman et al., 2008; Binici and Gunes, 2010). Many studies have found that genetic factors play an important role in the pathogenesis of osteoporosis (Jin and Ralston, 2001; Recker and Deng, 2002). It has been estimated that the heritability of osteoporosis-related traits (e.g., bone mineral density) can be as high as 60–80% (Uitterlinden et al., 2004). To date, dozens of risk genes for osteoporosis have been identified, of which ESR1, LRP4, ITGA1, LRP5, SOST, SPP1, TNFRSF11A, TNFRSF11B, and TNFSF11 are thought to be involved in bone mineral density (BMD) homeostasis, bone remodeling, and bone matrix composition, and thus influence BMD and osteoporotic fractures. In addition, a number of candidate genes have been investigated (COL1A1, TGFB1, TGFB3, and VDR), but no clear genome-wide significant association with osteoporosis has been demonstrated (Saccone et al., 2015).

The vitamin D receptor (VDR) gene is located on chromosome 12q13 (Seuter et al., 2016) and exerts various biological effects by mediating the downstream signaling 1,25-dihydroxycholecalciferol (1,25(OH)2D3) (Fang et al., 2003). In human monocytes, 1,25(OH)2D3 regulates chromatin susceptibility at 8979 loci (Ling et al., 2016), and as such, VDR single-nucleotide polymorphisms (SNPs) have been associated with various diseases, including reduced bone mineral density and osteoporosis (Gómez et al., 1999; Garnero et al., 2005). In recent years, numerous studies have reported the association of VDR polymorphisms (e.g., BsmI, ApaI, TaqI, FokI, and Cdx2) with osteoporotic fractures. However, these results were inconsistent and even conflicting. For example, Garnero et al. found that the VDR BsmI B allele was associated with lower BMD and an increased risk of fracture (Alvarez-Hernández et al., 2003). In contrast, other studies found no association between the B allele and the risk of osteoporotic fractures (Uitterlinden et al., 2001; Horst-Sikorska et al., 2005; Iván et al., 2008; Karpiński et al., 2017). Similarly, there were conflicting associations between the ApaI, TaqI, FokI, and Cdx2 polymorphisms and osteoporotic fractures in different studies (Gennari et al., 1999; Gómez et al., 1999; Garnero et al., 2005; Nguyen et al., 2005; Fang et al., 2006; Ji et al., 2010; Horst-Sikorska et al., 2013; Jawiarczyk-Przybyłowska et al., 2019; Iveta et al., 2020). These different results may be owing to differences in sample size, ethnicity, and sampling methods used. Although correlations between the VDR BsmI, ApaI, TaqI, and FokI polymorphisms and the risk of osteoporotic fracture development have been reported in several meta-analyses (Aerssens et al., 2000; Moher et al., 2009; Shen et al., 2014; Gao et al., 2015), there are some limitations in these studies. First, their findings are inconsistent; in the study of Ji et al., the bb genotype in the BsmI gene significantly reduced the risk of fracture (odds ratio (OR) 0.87, 95% confidence interval (CI): 0.76–0.98); in the grouped study, they found that the frequency of the bb genotype was significantly decreased in patients with hip fracture, and the frequency of the Tt genotype was also decreased in patients with hip fracture (Gao et al., 2015), while the frequency of the tt genotype was increased in patients with hip fracture. In addition, they observed an increase in the frequency of the Aa genotype in patients with vertebral fractures. Similarly, in a subgroup analysis, Gao et al. found that the BsmI gene was associated with osteoporotic fractures when the control group was population-derived (OR BB vs. bb 1.22, 95% CI 1.01–1.48; OR B vs. b 1.10, 95% CI 1.00–1.20) (Aerssens et al., 2000). No significant association was found between the BmsI and TaqI by Fang et al. and the BmsI by Shen et al. (BsmI OR 0.98, 95% CI 0.86–1.12; BsmI [b vs. B] OR 1.07, 95% CI 0.90–1.29; TaqI [T vs. t] OR 0.89, 95% CI 0.68–1.15; ApaI [A vs. a] OR 0.91, 95% CI 0.76–1.08; FokI [F vs. f] OR 1.20, 95% CI 0.76–1.90) (Moher et al., 2009; Shen et al., 2014). Second, a literature quality assessment was not performed in some of the meta-analyses (Shen et al., 2014; Gao et al., 2015). Finally, the Hardy–Weinberg equilibrium (HWE) test was not performed in the three studies (Moher et al., 2009; Shen et al., 2014; Gao et al., 2015), and not all studies on the VDR polymorphisms with osteoporosis fracture risk adjusted the P-value (Aerssens et al., 2000; Moher et al., 2009; Shen et al., 2014; Gao et al., 2015). Therefore, an updated meta-analysis was conducted to provide results that were more reliable regarding these issues.

## Materials and Methods

### Search Strategy

The present meta-analysis was performed based on the Preferred Reporting Items for Systematic Reviews and Meta-Analyses (PRISMA) checklist. The databases searched included PubMed, EMBASE, China Knowledge Network, and China Wanfang Data Knowledge Service Platform to analyze the relationship between VDR polymorphisms and osteoporotic fracture risk. The search strategy was (“vitamin D receptor” or “VDR”) and (“polymorphism” or “variant” or “variation” or “mutation” or “SNP” or “genome-wide association study” or “genetic association study” or “genotype” or “allele”) and (“Fractures, Bone” or “Broken Bones” or “Fractures” or “Fracture” or “Broken Bone” or “Bone Fractures” or “Bone Fracture”). The search deadline was March 2021.

### Selection Criteria

The inclusion criteria were as follows: 1) case–control or cohort studies; 2) investigation of the association between VDR BsmI, ApaI, TaqI, FokI, and Cdx2 polymorphisms and osteoporosis risk; and 3) detailed control and case group genotype data or their OR with 95% CI. The exclusion criteria were as follows: 1) overlapping studies; 2) articles without detailed genotype data; and 3) abstracts, case reports, editorials, reviews, letters, and meta-analyses.

A total of 221 articles were retrieved from all databases. In all, 194 articles were subsequently excluded because they were abstracts, case reports, editorials, reviews, letters, or meta-analyses. When the remaining 27 articles were read, two articles were excluded because patients with both osteoporosis and osteoporotic fractures were considered in the same group. In addition, two articles were found to be repetitive, and one article had missing genotype data, and attempts to contact the corresponding author have not been answered. In the end, 23 relevant studies were included. In the process of article screening, the retrieval work and the screening process were performed by Yi-yang Mu and Biao-Liu independently and then summarized, and the author Bin-Chen made the final decision when there was any disagreement.

### Data Extraction

We predesigned the data extraction form. The data from the selected articles were extracted and cross-checked according to the defined inclusion and exclusion criteria. When different results were obtained, and no consensus could be reached after the discussion, a third author was invited to repeat the data extraction and check for confirmation. If the data were unclear or questionable in the article, the author was contacted to obtain the original data. The following information was extracted: first author of the article, year of publication, country of study, corresponding continent, origin of cases and controls, type of osteoporotic fracture, sex of study subjects, number of cases and controls, number of genotypes distributed among cases and controls, diagnostic criteria for osteoporotic fractures, and conclusion of the investigators.

### Quality Assessment

The quality of all articles was independently assessed by two authors. We adopted and refined the quality assessment criteria from two previous meta-analyses (Aerssens et al., 2000; Moher et al., 2009). Supplementary Table S1 lists the quality assessment scales for studies on the factors associated with osteoporotic fracture risk. A total of 20 points were awarded, with articles scoring above 12 rated as excellent, those lying between 9 and 12 labeled as moderate, and studies scoring below 9 rated as poor.

### Statistical Analysis

The strength of association was evaluated using ORs with their 95% CIs and was considered statistically significant when the P-value was <0.05. Comparisons were performed using the following five genetic models: 1) allelic model, 2) additive model, 3) dominant model, 4) recessive model, and 5) over-dominant model. The chi-square–based Q test and I^2^ values were used to assess heterogeneity. *P* > 0.10 and/or I^2^ < 50% indicated no significant heterogeneity among the included studies, and a fixed-effects model was used. Otherwise, a random-effects model was applied. Publication bias was detected using Begg’s funnel plot and Egger’s test. Sensitivity analyses were assessed using three methods: 1) exclusion of one included study; ,2) exclusion of included HWD studies and low-quality studies, and 3) only including high-quality studies, the Hardy–Weinberg equilibrium (HWE), and matched studies. A chi-square goodness-of-fit test was applied to assess the HWE, and controls were identified as the HWE when *p* > 0.05. In addition, the false-positive reporting probability (FPRP) test and Venice criteria were used to assess the credibility of statistically significant associations. The abovementioned statistical analyses were made possible using Stata 12.0 software.

## Results

### Description of Included Studies

**FIGURE 1:**
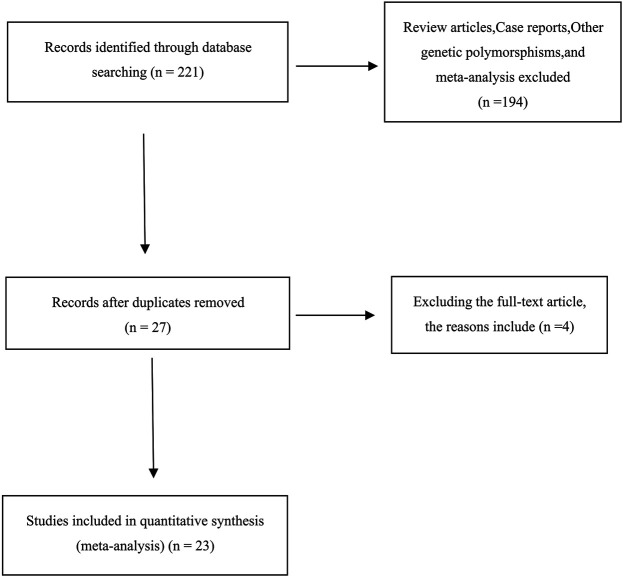
Flow diagram of the literature search.

A total of 221 relevant studies were retrieved, and 23 articles met our criteria (5,844 osteoporotic fracture cases and 19,339 controls), of which 18 articles examined VDR BsmI (involving 2,429 osteoporotic fracture cases and 5,187 controls), eight studies discussed VDR ApaI (involving 875 osteoporotic fracture cases and 2,120 controls), nine studies reported VDR TaqI (involving 860 osteoporotic fracture cases and 2,538 controls), seven studies documented VDR FokI (involving 579 osteoporotic fracture cases and 1635 controls), and three studies investigated VDR Cdx2 (involving 1101 osteoporotic fracture cases and 7859 controls), and how each of these polymorphisms correlates with osteoporotic fracture risk. In addition, 18, 5, and 1 case–control studies have been conducted in European, American, and Asian populations, respectively. Among them, four studies discussed these associations in men, and 22 studies analyzed these relationships in women. Finally, there were six high-quality studies and 12 medium-quality studies discussing VDR BsmI; two high-quality studies and seven medium-quality studies discussing VDR ApaI; two high-quality studies and six medium-quality studies on VDR TaqI; one high-quality, five medium-quality, and one low-quality studies on VDR FokI; and one medium-quality and two low-quality studies on VDR Cdx2. Table 1 shows the detailed characteristics and scores of each study. The literature selection and inclusion processes are illustrated in Figure 1. Tables 2–6 show the genotype frequencies of the VDR BsmI, ApaI, TaqI, FokI, and Cdx2 polymorphisms, and the impact of each on the risk of osteoporotic fracture.

**TABLE 1:** Main characteristics and Quality score of studies included.

First author / Year	Country	Ethnicity	Gender	Cases					Controls					score
				N	Age^a^	OF site	Diagnosis	Matching	N	Age^a^	HWE	Healthy	BMD site	
Houston et al. (1996)	UK	E	Female	44	66.0 ± 0.85	Verterbral	WHO	Age and Sex	44	65.3 ± 0.95	HWE	Yes	Verterbral	11
Feskanich et al. (1998)	USA	Am	Female	54	62.3±5.7	Hip	WHO	Age and Sex	108	62.2±5.7	HWE	Yes	Hip	13
Feskanich et al. (1998)	USA	Am	Female	163	58.3±6.8	Forearm	WHO	Age and Sex	163	58.1±6.7	HWE	Yes	Forearm	13
Ramalho et al. (1998)	Brazil	Am	Female	56	78.52 ± 7.2	Hip	Ne	Age and Sex	36	78.52 ± 7.2	HWE	Yes	Hip	11
Gómez et al. (1999)	Spain	E	Female	37	Ne	Verterbral	WHO	Sex	122	Ne	HWE	Yes	Verterbral	13
Gómez et al. (1999)	Spain	E	Men	39	Ne	Verterbral	WHO	Sex	114	Ne	HWE	Yes	Verterbral	13
Aerssens et al. (2000)	Belgium	E	Female	135	78±9	Hip	WHO	Age and Sex	239	76±4	HWE	Yes	Hip	11
Langdahl et al. (2000)	Denmark	E	Female	80	64.8 ± 8.3	Verterbral	WHO	Sex	80	47.2 ±13.6	HWE	Yes	Verterbral	12
Langdahl et al. (2000)	Denmark	E	Men	30	55.7 ± 11.0	Verterbral	WHO	Age and Sex	73	51.1 ± 15.7	HWE	Yes	Verterbral	13
Välimäki et al. (2001)	Finland	E	Female	64	Ne	Verterbral	WHO	Sex	108	Ne	HWE	Yes	Verterbral	11
Uitterlinden et al. (2001)	Netherlands	E	Female	97	66.4 ± 7.0	Ne	WHO	Age and Sex	907	66.4 ± 7.0	HWE	Yes	Ne	15
Alvarez-Hernández et al. (2003)	Spain	E	Men	20	64 ±9	Verterbral	Ne	Age and Sex	134	64±9	HWE	Yes	Verterbral	13
Garnero et al. (2005)	France	E	Female	86	Ne	Noverterbral	WHO	Sex	589	Ne	HWE	Yes	N/A	15
Garnero et al. (2005)	France	E	Female	34	Ne	Verterbral	WHO	Sex	589	Ne	HWE	Yes	Verterbral	15
Horst-Sikorska et al. (2005)	Poland	E	Female	48	Ne	Ne	WHO	Sex	93	Ne	HWE	Yes	Ne	11
Efesoy et al. (2003)	Turkey	A	Female	18	65.75±9.8	Verterbral	T-Score < 2.0	Age and Sex	74	62.4±8.7	HWE	Yes	Verterbral	10
Wengreen et al. (2006)	USA	Am	Female	819	76.7±9.1	Hip	WHO	Age and Sex	854	76±9.4	HWD	Yes	Hip	11
Horst-Sikorska et al. (2007)	Poland	E	Female	85	64.4 ± 10.9	Vertebral and femur	WHO	Age and Sex	191	65.5 ± 9.9	HWE	Yes	Vertebral and femur	12
Quevedo et al. (2008)	Chile	Am	Female	67	77 ± 4	Hip	T-Score < 2.0	Age and Sex	59	78 ± 9	HWD	Yes	Hip	10
Horst-Sikorska et al. (2013)	Poland	E	Female	167	68.5 ± 8.2	Verterbral	WHO	Age and Sex	216	63.5 ± 9.1	HWE	Yes	Verterbral	13
Horst-Sikorska et al. (2013)	Poland	E	Female	117	68.5 ± 8.2	Noverterbral	WHO	Age and Sex	216	63.5 ± 9.1	HWE	Yes	N/A	13
Karpinski et al. (2017)	Brazil	E	Ne	100	11.5±2.5	Ne	WHO	Age and Sex	127	13.5±2.5	HWE	Yes	Ne	10
Aleksandra et al. (2019)	Poland	E	Ne	69	60.3 ± 11.2	Hip	WHO	Age and Sex	51	56.7 ± 11.2	HWE	Yes	Hip	9
Langdahl et al. (2000)	Denmark	E	Female	78	64.8 ± 8.3	Verterbral	WHO	Sex	74	47.2 ± 13.6	HWE	Yes	Verterbral	12
Langdahl et al. (2000)	Denmark	E	Men	29	55.7 ± 11.0	Verterbral	WHO	Age and Sex	73	51.1 ± 15.7	HWE	Yes	Verterbral	13
Uitterlinden et al. (2001)	Netherlands	E	Female	97	66.4 ± 7.0	Ne	WHO	Age and Sex	907	66.4 ± 7.0	HWE	Yes	Ne	15
Alvarez-Hernández et al. (2003)	Spain	E	Men	17	65 ±9	Verterbral	Ne	Age and Sex	117	65 ±9	HWE	Yes	Verterbral	13
Horst-Sikorska et al. (2005)	Poland	E	Female	48	Ne	Verterbral	WHO	Sex	93	Ne	HWE	Yes	Verterbral	11
Horst-Sikorska et al. (2007)	Poland	E	Female	85	64.4 ± 10.9	Hip	WHO	Age and Sex	191	65.5 ± 9.9	HWE	Yes	Hip	12
Quevedo et al. (2008)	Chile	Am	Female	67	77 ± 4	Hip	T-Score < 2.0	Age and Sex	59	78 ± 9	HWE	Yes	Hip	10
Horst-Sikorska et al. (2013)	Poland	E	Female	168	68.5 ± 8.2	Verterbral	WHO	Age and Sex	216	63.5 ± 9.1	HWE	Yes	Verterbral	13
Horst-Sikorska et al. (2013)	Poland	E	Female	117	68.5 ± 8.2	Noverterbral	WHO	Age and Sex	216	63.5 ± 9.1	HWE	Yes	N/A	13
Karpinski et al. (2017)	Brazil	E	Ne	100	11.5±2.5	Ne	WHO	Age and Sex	123	13.5±2.5	HWE	Yes	Ne	10
Aleksandra et al. (2019)	Poland	E	Ne	69	60.3 ± 11.2	Hip	WHO	Age and Sex	51	56.7 ± 11.2	HWE	Yes	Hip	9
Langdahl et al. (2000)	Denmark	E	Female	78	64.8± 8.3	Verterbral	WHO	Sex	75	47.2± 13.6	HWE	Yes	Verterbral	12
Langdahl et al. (2000)	Denmark	E	Men	29	55.7±11.0	Verterbral	WHO	Age and Sex	73	51.1 ± 15.7	HWE	Yes	Verterbral	13
Uitterlinden et al. (2001)	Netherlands	E	Female	97	66.4 ± 7.0	Ne	WHO	Age and Sex	907	66.4±7.0	HWE	Yes	Ne	15
Alvarez-Hernández et al. (2003)	Spain	E	Men	21	64 ±9	Verterbral	WHO	Age and Sex	117	64 ±9	HWE	Yes	Verterbral	13
Horst-Sikorska et al. (2005)	Poland	E	Female	48	Ne	Verterbral	WHO	Sex	93	Ne	HWE	Yes	Verterbral	11
Nguyen et al. (2005)	Australia	E	Female	69	Ne	Hip	WHO	Sex	608	Ne	HWE	Yes	Hip	12
Quevedo et al. (2008)	Chile	Am	Female	67	77 ± 4	Hip	T-Score < 2.0	Age and Sex	59	78 ± 9	HWE	Yes	Hip	10
Horst-Sikorska et al. (2013)	Poland	E	Female	168	68.5 ± 8.2	Verterbral	WHO	Age and Sex	216	63.5 ± 9.1	HWD	Yes	Verterbral	13
Horst-Sikorska et al. (2013)	Poland	E	Female	117	68.5 ± 8.2	Noverterbral	WHO	Age and Sex	216	63.5 ± 9.1	HWD	Yes	N/A	13
Karpinski et al. (2017)	Brazil	E	Ne	97	11.5±2.5	Ne	WHO	Age and Sex	123	13.5±2.5	HWD	Yes	Ne	10
Aleksandra et al. (2019)	Poland	E	Ne	69	60.3 ± 11.2	Hip	WHO	Age and Sex	51	56.7 ± 11.2	HWE	Yes	Hip	9
Gennari et al. (1999)	Belgium	E	Female	68	Ne	Verterbral	WHO	Sex	332	Ne	HWE	Yes	Verterbral	11
Langdahl et al. (2000)	Denmark	E	Female	79	64.8 ± 8.3	Verterbral	WHO	Sex	80	47.2 ± 13.6	HWE	Yes	Verterbral	12
Langdahl et al. (2000)	Denmark	E	Men	30	55.7±11.0	Verterbral	WHO	Age and Sex	73	51.1±15.7	HWE	Yes	Verterbral	13
Horst-Sikorska et al. (2007)	Poland	E	Female	85	64.4 ± 10.9	Hip	WHO	Age and Sex	191	65.5 ± 9.9	HWE	Yes	Hip	12
Quevedo et al. (2008)	Chile	Am	Female	67	77 ± 4	Hip	T-Score < 2.0	Age and Sex	59	78 ± 9	HWE	Yes	Hip	10
Karpinski et al. (2017)	Brazil	E	Ne	100	11.5±2.5	Ne	WHO	Age and Sex	124	13.5±2.5	HWD	Yes	Ne	10
Aleksandra et al. (2019)	Poland	E	Ne	69	60.3 ± 11.2	Hip	WHO	Age and Sex	51	56.7 ± 11.2	HWE	Yes	Hip	9
Iveta et al. (2020)	Slovak	E	Female	13	67.16 ± 9.22	Verterbral	Ne	Age and Sex	390	65.01 ± 9.28	HWE	Yes	Verterbral	8
Iveta et al. (2020)	Slovak	E	Female	68	67.16 ± 9.22	Noverterbral	Ne	Age and Sex	335	65.01 ± 9.28	HWE	Yes	N/A	8
Fang et al. (2003)	Dutch	E	Ne	381	Ne	Ne	WHO	Sex	1534	Ne	HWE	Yes	Ne	12
Fang et al. (2003)	Dutch	E	Ne	217	Ne	Verterbral	WHO	Sex	1698	Ne	HWE	Yes	Verterbral	12
Fang et al. (2003)	Dutch	E	Ne	248	Ne	Noverterbral	WHO	Sex	2600	Ne	HWD	Yes	N/A	12
Ling et al. (2016)	China	A	Female	67	Ne	Noverterbral	WHO	Sex	361	Ne	HWE	Yes	N/A	11
Ling et al. (2016)	China	A	Men	15	Ne	Noverterbral	WHO	Sex	295	Ne	HWE	Yes	N/A	11
Ling et al. (2016)	China	A	Female	76	Ne	Ne	WHO	Sex	352	Ne	HWE	Yes	Ne	11
Ling et al. (2016)	China	A	Men	16	Ne	Ne	WHO	Sex	294	Ne	HWE	Yes	Ne	11
Iveta et al. (2020)	Slovak	E	Female	13	67.16 ± 9.22	Verterbral	Ne	Age and Sex	390	65.01 ± 9.28	HWE	Yes	Verterbral	8
Iveta et al. (2020)	Slovak	E	Female	68	67.16 ± 9.22	Noverterbral	Ne	Age and Sex	335	65.01 ± 9.28	HWE	Yes	N/A	8

Ne = not available : N/A=Non-vertebral fractures; OF = Osteoporotic fracture;

a 1= **(**Mean±SD) yrs;HWE: Hardy-Weinberg equilibrium;HWD :Hardy Weinberg Disequilibrium

**TABLE 2:** Genotype frequencies of VDR BsmI polymorphism in studies included in this meta-analysis.

First author/year	Country	Ethnicity	Source of controls	Fracture type	Sex	HWE		Number of samples			Genotypes of cases			Alleles of cases		Minor allele frequency	Genotypes of controls			Controls' alleles		Minor allele frequency
						chi2	*P*	Cases	Controls	Total	B/B	B/b	b/b	B	b		B/B	B/b	b/b	B	b	
Houston et al. (1996)	United Kingdom	E	Hospital	Vertebral	F	0.571	0.4498	44	44	88	8	19	17	35	53	1.514285714	9	19	16	37	51	1.378378378
Feskanich et al. (1998)	United States	Am	Population	Hip	F	0.085	0.7702	54	108	162	16	21	17	53	55	1.037735849	16	53	39	85	131	1.541176471
Feskanich et al. (1998)	United States	Am	Population	Forearm	F	2.055	0.1517	163	163	326	25	83	55	133	193	1.45112782	26	89	48	141	185	1.312056738
Ramalho et al. (1998)	Brazil	Am	Hospital	Hip	F	3.825	0.0505	56	36	92	13	23	20	49	63	1.285714286	7	11	18	25	47	1.88
Gómez et al. (1999)	Spain	E	Population	Vertebral	F	1.377	0.2407	37	122	159	7	20	10	34	40	1.176470588	20	51	51	91	153	1.681318681
Gómez et al. (1999)	Spain	E	Population	Vertebral	M	0.283	0.5945	39	114	153	6	18	15	30	48	1.6	18	58	38	94	134	1.425531915
Aerssens et al. (2000)	Belgium	E	Hospital	Hip	F	0.547	0.4594	135	239	374	26	60	49	112	158	1.410714286	52	125	62	229	249	1.087336245
Langdahl BL et al. (2000)	Denmark	E	Community	Vertebral	F	1.749	0.1860	80	80	160	23	38	19	84	76	0.904761905	25	34	21	84	76	0.904761905
Langdahl BL et al. (2000)	Denmark	E	Community	Vertebral	M	2.893	0.0890	30	73	103	8	16	6	32	28	0.875	15	28	30	58	88	1.517241379
Välimäki et al. (2001)	Finland	E	Hospital	Vertebral	F	1.307	0.2529	64	108	172	9	35	20	53	75	1.41509434	10	54	44	74	142	1.918918919
Uitterlinden et al. (2001)	Netherlands	E	Population	Any	F	3.045	0.0810	97	907	1004	7	41	49	55	139	2.527272727	172	416	319	760	1054	1.386842105
Alvarez-Hernández et al. (2003)	Spain	E	Population	Verterbral	M	0.248	0.6183	20	134	154	3	9	8	15	25	1.666666667	22	68	44	112	156	1.392857143
Garnero et al. (2005)	France	E	Population	Non-vertebral	F	0.140	0.7082	86	589	674	20	46	20	86	86	1	90	286	213	466	712	1.527896996
Garnero et al. (2005)	France	E	Population	Vertebral	F	0.140	0.7082	34	589	623	5	16	13	26	42	1.615384615	90	286	213	466	712	1.527896996
Horst-Sikorska et al. (2005)	Poland	E	Population	Any	F	1.539	0.2147	48	93	141	3	19	26	25	71	2.84	18	39	36	75	111	1.48
Efesoy et al. (2003)	Turkey	A	Hospital	Vertebral	F	2.206	0.1375	18	74	92	0	10	8	10	26	2.6	12	43	19	67	81	1.208955224
Wengreen et al. (2006)	United States	Am	Hospital	Hip	F	4.115	**0.0425**	819	854	1673	154	393	272	701	937	1.336661912	140	376	338	656	1052	1.603658537
Horst-Sikorska et al. (2007)	Poland	E	Hospital	Vertebral and femur	F	0.913	0.3394	85	191	276	10	35	40	55	115	2.090909091	33	85	73	151	231	1.529801325
Quevedo et al. (2008)	Chile	Am	Hospital	Hip	F	3.989	**0.0458**	67	59	126	11	46	10	68	66	0.970588235	9	37	13	55	63	1.145454545
Horst-Sikorska et al. (2013)	Poland	E	Hospital	Vertebral	F	2.240	0.1345	167	216	383	27	80	60	134	200	1.492537313	42	94	80	178	254	1.426966292
Horst-Sikorska et al. (2013)	Poland	E	Hospital	Non-vertebral	F	2.240	0.1345	117	216	333	13	51	53	77	157	2.038961039	42	94	80	178	254	1.426966292
Karpiński et al. (2017)	Brazil	E	Hospital	Any	M/F	0.299	0.5846	100	127	227	8	49	43	65	135	2.076923077	19	64	44	102	152	1.490196078
Aleksandra et al. (2019)	Poland	E	Hospital	HIp	M/F	1.051	0.3053	69	51	120	32	26	11	90	48	0.533333333	20	21	10	61	41	0.672131148

**TABLE 3:** Genotype frequencies of VDR ApaI polymorphism in studies included in this meta-analysis.

First author/year	Country	Ethnicity	Source of controls	Fracture type	Sex	HWE		Number of samples			Genotypes of cases			Alleles of cases		Minor allele frequency	Genotypes of controls			Controls’ alleles		Minor allele frequency
						chi2	pr	Cases	Controls	Total	A/A	A/a	a/a	A	a		A/A	A/a	a/a	A	a	
Langdahl et al. (2000)	Denmark	E	Community	Vertebral	F	1.155	0.2826	78	74	152	22	44	12	88	68	0.772727273	25	32	17	82	66	0.804878049
Langdahl et al. (2000)	Denmark	E	Community	Vertebral	M	1.779	0.1823	29	73	102	8	17	4	33	25	0.757575758	18	42	13	78	68	0.871794872
Uitterlinden et al. (2001)	Netherlands	E	Population	Any	F	2.709	0.0998	97	907	1004	15	48	34	78	116	1.487179487	258	428	221	944	870	0.921610169
Alvarez-Hernández et al. (2003)	Spain	E	Population	Vertebral	M	0.118	0.7313	17	117	134	4	12	1	20	14	0.7	33	60	24	123	108	0.87804878
Horst-Sikorska et al. (2005)	Poland	E	Population	Vertebral	F	1.445	0.2293	48	93	141	8	21	19	37	59	1.594594595	24	52	17	100	86	0.86
Horst-Sikorska W et al. (2007)	Poland	E	Population	Hip	F	0.450	0.5024	85	191	276	20	36	29	76	74	0.973684211	49	100	42	198	184	0.929292929
Quevedo et al. (2008)	Chile	Am	Hospital	Hip	F	0.383	0.5363	67	59	126	25	31	11	81	53	0.654320988	18	27	14	63	55	0.873015873
Horst-Sikorska et al. (2013)	Poland	E	Hospital	Vertebral	F	1.508	0.2195	168	216	384	41	83	44	165	171	1.036363636	48	117	51	213	219	1.028169014
Horst-Sikorska et al. (2013)	Poland	E	Hospital	Non-vertebral	F	1.508	0.2195	117	216	333	18	59	40	95	139	1.463157895	48	117	51	213	219	1.028169014
Karpinski et al. (2017)	Brazil	E	Hospital	Any	M/F	0.204	0.6516	100	123	223	23	43	34	89	111	1.247191011	29	64	30	122	124	1.016393443
Aleksandra et al. (2019)	Poland	E	Hospital	Hip	M/F	0.157	0.6916	69	51	120	17	35	17	69	69	1	15	24	12	54	48	0.888888889

**TABLE 4:** Genotype frequencies of VDR TaqI polymorphism in studies included in this meta-analysis.

First author/year	Country	Ethnicity	Source of controls	Fracture type	Sex	HWE		Number of samples			Genotypes of cases			Alleles of cases		Minor allele frequency	Genotypes of controls			Controls' alleles		Minor allele frequency
						chi2	Pr	Cases	Controls	Total	T/T	T/t	t/t	T	t		T/T	T/t	t/t	T	t	
Langdahl et al. (2000)	Denmark	E	Community	Vertebral	F	0.231	0.6304	78	75	153	23	41	14	87	69	0.793103448	28	34	13	90	60	0.666666667
Langdahl et al. (2000)	Denmark	E	Community	Vertebral	M	0	0.9945	29	73	102	8	19	2	35	23	0.657142857	29	34	10	92	54	0.586956522
Uitterlinden et al. (2001)	Netherlands	E	Population	Any	F	3.045	0.081	97	907	1004	49	41	7	139	55	0.395683453	319	416	172	1054	760	0.721062619
Alvarez-Hernández et al. (2003)	Spain	E	Population	Vertebral	M	0.523	0.4695	21	117	138	7	7	7	21	21	1	40	60	17	140	94	0.671428571
Horst-Sikorska et al. (2005)	Poland	E	Population	Vertebral	F	2.554	0.11	48	93	141	26	19	3	71	25	0.352112676	38	37	18	113	73	0.646017699
Nguyen et al. (2005)	Australia	E	Population	Hip	F	1.023	0.3119	69	608	677	24	27	18	75	63	0.84	218	302	88	738	478	0.647696477
Quevedo et al. (2008)	Chile	Am	Hospital	Hip	F	1.912	0.1668	67	59	126	26	31	10	83	51	0.614457831	17	34	8	68	50	0.735294118
Horst-Sikorska et al. (2013)	Poland	E	Hospital	Vertebral	F	4.237	**0.0396**	168	216	384	62	79	27	203	133	0.655172414	82	90	44	254	178	0.700787402
Horst-Sikorska et al. (2013)	Poland	E	Hospital	Non-vertebral	F	4.237	**0.0396**	117	216	333	55	49	13	159	75	0.471698113	82	90	44	254	178	0.700787402
Karpinski et al. (2017)	Brazil	E	Hospital	Any	M/F	21.417	**0**	97	123	220	43	52	2	138	56	0.405797101	44	77	2	165	81	0.490909091
Aleksandra et al. (2019)	Poland	E	Hospital	Hip	M/F	0.462	0.4968	69	51	120	32	26	11	90	48	0.533333333	20	22	9	62	40	0.64516129

**TABLE 5:** Genotype frequencies of VDR FokI polymorphism in studies included in this meta-analysis.

First author/year	Country	Ethnicity	Source of controls	Fracture type	Sex	HWE		Number of samples			Genotypes of cases			Alleles of cases		Minor allele frequency	Genotypes of controls			Controls' alleles		Minor allele frequency
						chi2	pr	Cases	Controls	Total	F/F	F/f	f/f	F	f		F/F	F/f	f/f	F	f	
Gennari et al. (1999)	Belgium	E	Hospital	Vertebral	F	0.373	0.5413	68	332	400	21	30	17	72	64	0.888888889	138	156	38	432	232	0.537037037
Langdahl et al. (2000)	Denmark	E	Community	Vertebral	F	2.554	0.11	79	80	159	28	41	10	97	61	0.628865979	34	31	15	99	61	0.616161616
Langdahl et al. (2000)	Denmark	E	Community	Vertebral	M	0.018	0.8943	30	73	103	12	13	5	37	23	0.621621622	30	34	9	94	52	0.553191489
Horst-Sikorska W et al. (2007)	Poland	E	Population	Hip	F	1.743	0.1868	85	191	276	40	35	10	115	55	0.47826087	76	82	33	234	148	0.632478632
Quevedo et al. (2008)	Chile	Am	Hospital	Hip	F	0.107	0.744	67	59	126	29	27	11	85	49	0.576470588	27	25	7	79	39	0.493670886
Karpinski et al. (2017)	Brazil	E	Hospital	Any	M/F	7.538	**0.006**	100	124	224	26	49	25	101	99	0.98019802	32	76	16	140	108	0.771428571
Aleksandra et al. (2019)	Poland	E	Hospital	Hip	M/F	0.042	0.8383	69	51	120	27	32	10	86	52	0.604651163	18	24	9	60	42	0.7
Iveta et al. (2020)	Slovak	E	Hospital	Vertebral	F	0.205	0.6505	13	390	403	1	9	3	11	15	1.363636364	86	199	105	371	409	1.102425876
Iveta et al. (2020)	Slovak	E	Hospital	Non-vertebral	F	0.507	0.4766	68	335	403	5	34	29	44	92	2.090909091	82	174	79	338	332	0.982248521

**TABLE 6:** Genotype frequencies of VDR Cdx2 polymorphism in studies included in this meta-analysis.

First author/year	Country	Ethnicity	Source of controls	Fracture type	Sex	HWE		Number of samples			Genotypes of cases			Alleles of cases		Minor allele frequency	Genotypes of controls			Controls' alleles		Minor allele frequency
						chi2	pr	Cases	Controls	Total	G/G	A/G	A/A	G	A		G/G	A/G	A/A	G	A	
Fang et al. (2003)	Netherlands	E	Hospital	Any	F/M	2.293	0.13	381	1534	1915	268	103	10	639	123	0.192488263	1002	464	68	2468	600	0.243111831
Fang et al. (2003)	Netherlands	E	Hospital	Vertebral	F/M	2.159	0.1417	217	1698	1915	156	56	5	368	66	0.179347826	1114	511	73	2739	657	0.239868565
Fang et al. (2003)	Netherlands	E	Hospital	Non-vertebral	F/M	4.547	**0.033**	248	2600	2848	173	70	5	416	80	0.192307692	1721	768	111	4210	990	0.235154394
Ling et al. (2016)	China	A	Hospital	Non-vertebral	F	1.427	0.2323	67	361	428	15	35	17	65	69	1.061538462	130	164	67	424	298	0.702830189
Ling et al. (2016)	China	A	Hospital	Non-vertebral	M	0.595	0.4405	15	295	310	8	6	1	22	8	0.363636364	93	151	51	337	253	0.75074184
Ling et al. (2016)	China	A	Hospital	Any	F	1.140	0.2857	76	352	428	19	38	19	76	76	1	126	161	65	413	291	0.704600484
Ling et al. (2016)	China	A	Hospital	Any	M	0.510	0.475	16	294	310	8	7	1	23	9	0.391304348	93	150	51	336	252	0.75
Iveta et al. (2020)	Slovak	E	Hospital	Vertebral	F	0.001	0.9708	13	390	403	7	6	0	20	6	0.3	260	117	13	637	143	0.224489796
Iveta et al. (2020)	Slovak	E	Hospital	Non-vertebral	F	1.259	0.2619	68	335	403	21	38	9	80	56	0.7	246	85	4	577	93	0.16117851

### Meta-Analysis Results

**FIGURE 2:**
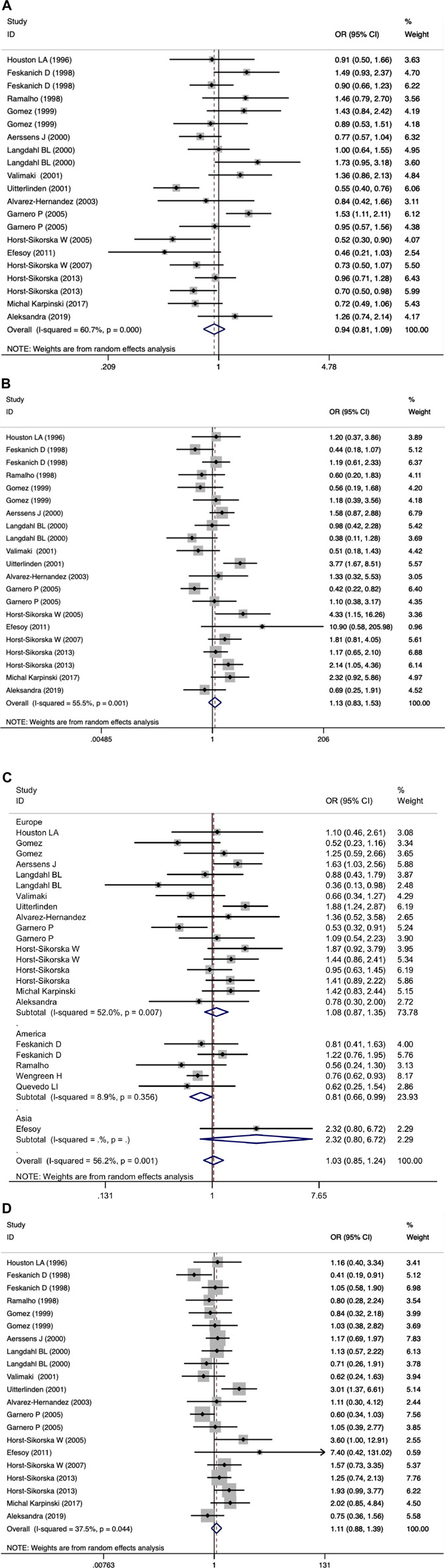
Forest plots of all selected studies on the association between VDR Bsml polymorphism and the risk of osteoporotic fracture in different races [**(A)** allele model, **(B)** additive model, **(C)** dominant model, and **(D)** recessive model].

We did not observe a significant association between the VDR BsmI polymorphism and the risk of osteoporotic fractures (*p* > 0.05) in all genetic models. However, subgroup analysis by race showed that the VDR BsmI B allele increased the risk of osteoporotic fracture (OR 1.17, 95% CI 1.03–1.34), and the BB genotype (additive model: OR 0.74, 95% CI 0.58–0.94; recessive model: OR 0.81, 95% CI 0.66–0.99) reduced the risk of osteoporotic fractures in Americans. We believe that articles with HWD control data should be excluded because the inclusion of HWD articles may interfere with the real results. When HWD-related article data were excluded, the positive results of the subgroup analysis corresponding to race changed. Table 7 summarizes the evaluation of the association between VDR BsmI polymorphism and the risk of osteoporotic fractures. Overall, the VDR BsmI polymorphism did not significantly increase the risk of osteoporotic fractures, as shown in Figure 2.

**TABLE 7:** Pooled estimates of association of VDR BsmI polymorphism and the risk of osteoporotic fracture.

Genetic model	Variable	Test of association		Tests for heterogeneity		Egger’s test
		OR (95% CI)	*P*	*P*_*h*_	*I* ^*2*^ (%)	*P*_*E*_
B vs b	Overall	0.94 (0.81–1.09)	0.413	<0.001	60.70	0.450
	Europe	0.92 (0.78–1.09)	0.322	<0.001	61.50	
	America	1.18 (0.82–1.70)	0.363	0.139	49.4	
	Female	0.92 (0.77–1.10)	0.369	<0.001	65.60	
	Male	1.09 (0.69–1.71)	0.709	0.183	41.1	
bb vs BB	Overall	1.13 (0.83–1.53)	0.437	<0.001	55.50	0.953
	Europe	1.20 (0.86–1.67)	0.289	0.002	56.20	
	America	0.73 (0.38–1.40)	0.347	0.186	40.5	
	Female	1.16 (0.81–1.65)	0.417	<0.001	61.10	
	Male	0.83 (0.38–1.82)	0.642	0.295	18.00	
Bb + bb vs BB	Overall	1.11 (0.88–1.39)	0.381	0.044	37.50	0.399
	Europe	1.18 (0.93–1.49)	0.171	0.100	32.10	
	America	0.72 (0.40–1.31)	0.284	0.182	41.3	
	Female	1.13 (0.86–1.48)	0.377	0.020	47.0	
	Male	0.91 (0.49–1.69)	0.756	0.823	0.00	
bb vs BB + Bb	Overall	1.08 (0.89–1.31)	0.457	0.007	48.40	0.098
	Europe	1.08 (0.87–1.35)	0.471	0.007	52.00	
	America	0.91 (0.59–1.42)	0.690	0.245	28.90	
	Female	1.09 (0.87–1.36)	0.449	0.006	53.50	
	Male	0.88 (0.40–1.95)	0.756	0.098	57.00	
BB + bb vs Bb	Overall	1.01 (0.89–1.15)	0.819	0.900	0.00	0.372
	Europe	0.99 (0.87–1.14)	0.935	0.893	0.00	
	America	1.13 (0.39–3.13)	0.545	0.299	17.10	
	Female	1.01 (0.88–1.16)	0.857	0.829	0.00	
	Male	0.95 (0.56–1.60)	0.846	0.305	15.80	

**VDR BsmI**: allele model: B vs. b, additive model: bb vs. BB, dominant model: Bb + bb vs. BB, recessive model: bb vs. BB+ Bb, over-dominant model: BB+ bb vs. Bb.

**FIGURE 3:**
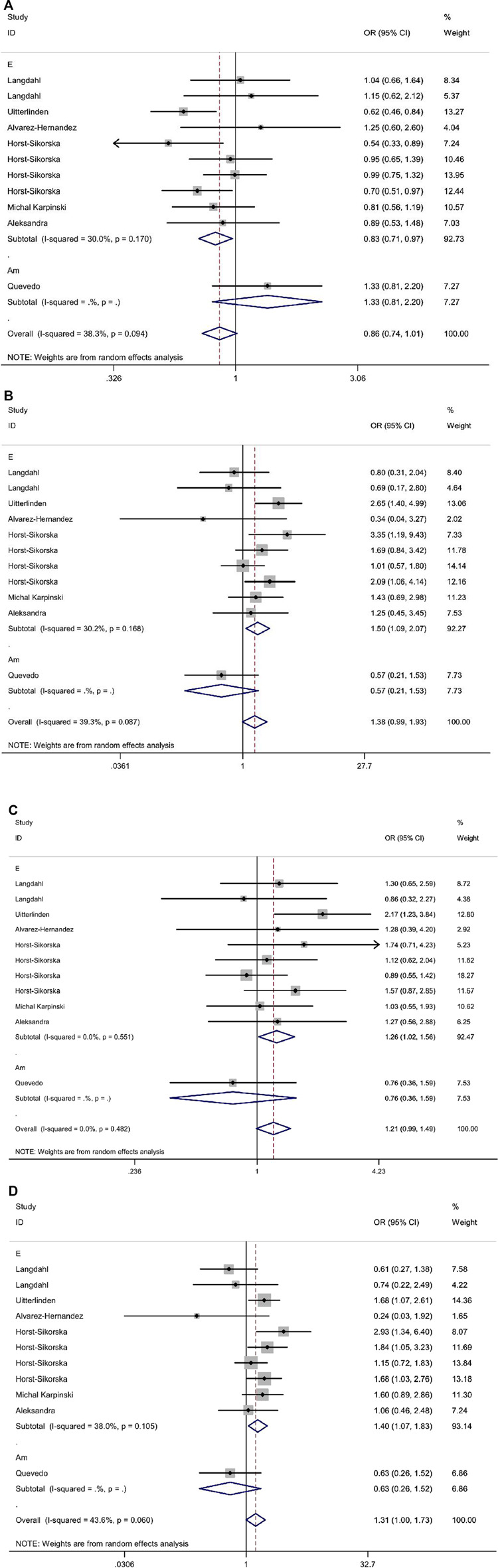
Forest plots of all selected studies on the association between VDR Apal polymorphism and the risk of osteoporotic fracture in different races [**(A)** allele model, **(B)** additive model, **(C)** dominant model, and **(D)** recessive model].

In the overall analysis, it was not found whether VDR ApaI polymorphism could significantly increase the risk of osteoporotic fracture (*p* > 0.05 in all genetic models). When stratified by race, the results showed that in the European population, the aa genotype increased the risk of osteoporotic fracture compared with the AA genotype (allelic model: OR 0.83, 95% CI 0.71–0.97; additive model: OR 1.50, 95% CI 1.09–2.07; dominant model: OR 1.26, 95% CI 1.02–1.56; recessive model: OR 1.40, 95% CI 1.07–1.83). All data are shown in Table 8 and Figure 3.

**TABLE 8:** Pooled estimates of association of VDR ApaI polymorphism and the risk of osteoporotic fracture.

Genetic model	Variable	Test of association		Tests for heterogeneity		Egger’s test
		OR (95% CI)	*P*	*P*_*h*_	*I* ^*2*^ (%)	*P*_*E*_
A vs a	Overall	0.86 (0.74–1.01)	0.072	0.094	38.30	0.220
	Europe	**0.83 (0.71–0.97)**	0.019	0.170	30.00	
	Female	0.84 (0.67–1.04)	0.104	0.031	56.90	
	Male	1.19 (0.75–1.91)	0.462	0.859	0	
aa vs AA	Overall	1.38 (0.99–1.93)	0.057	0.087	39.30	0.186
	Europe	**1.50 (1.09–2.07)**	0.012	0.168	30.20	
	Female	1.50 (0.97–2.32)	0.068	0.034	55.90	
	Male	0.57 (0.17–1.87)	0.353	0.604	0	
Aa + aa vs AA	Overall	1.21 (0.99–1.49)	0.063	0.482	0	0.947
	Europe	**1.26 (1.02–1.56)**	0.032	0.551	0	
	Female	1.27 (0.95–1.69)	0.103	0.192	30.90	
	Male	1.01 (0.47–2.14)	0.986	0.613	0	
aa vs AA + Aa	Overall	1.31 (1.00–1.73)	0.054	0.060	43.60	0.061
	Europe	**1.40 (1.07–1.83)**	0.015	0.105	38.00	
	Female	1.39 (0.99–1.94)	0.056	<0.040	54.60	
	Male	0.56 (0.19–1.58)	0.271	0.353	0	
AA + aa vs Aa	Overall	1.08 (0.90–1.30)	0.420	0.319	13.10	0.215
	Europe	1.08 (0.88–1.33)	0.443	0.248	21.10	
	Female	1.10 (0.88–1.36)	0.398	0.289	18.50	
	Male	0.70 (0.33–1.48)	0.349	0.277	15.20	

**VDR ApaI:** allele model: A vs. a, additive model: aa vs. AA, dominant model: Aa + aa vs. AA, recessive model: aa vs. AA + Aa, over-dominant model: AA+ aa vs. Aa.

Bold values represent with statistical significance.

**FIGURE 4:**
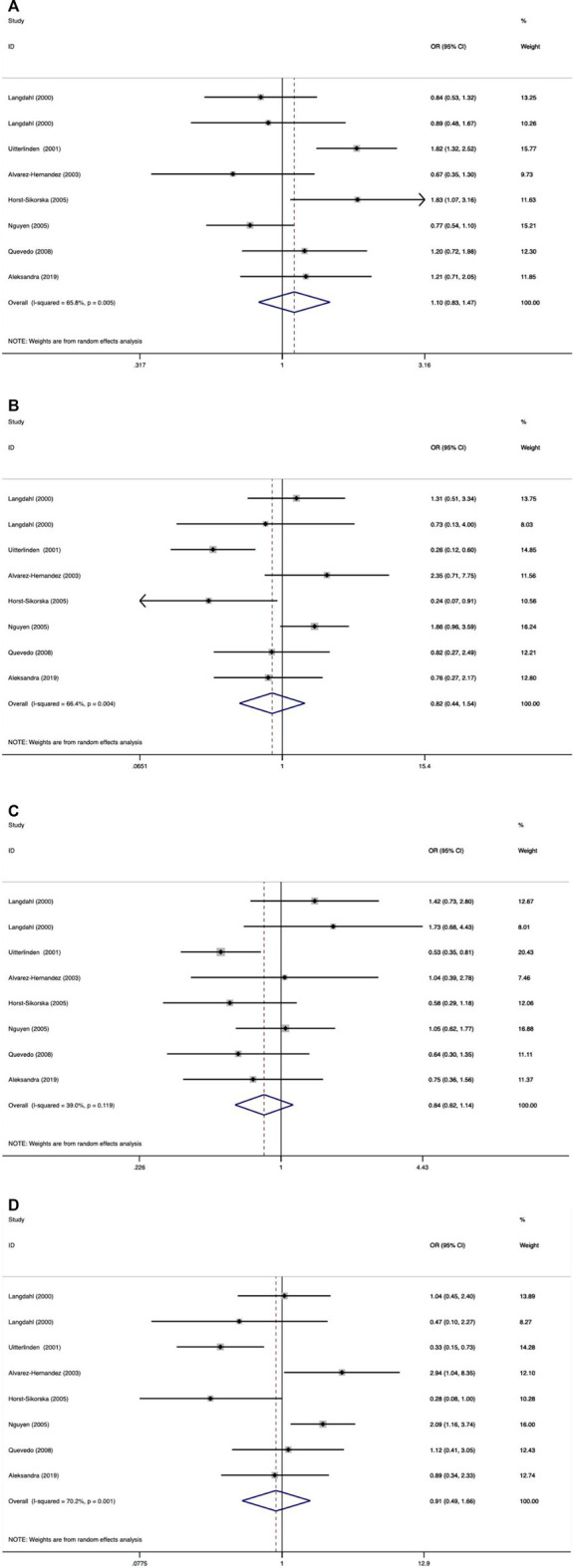
Forest plots of all selected studies on the association between VDR Taql polymorphism and the risk of osteoporotic fracture in different races [**(A)** allele model, **(B)** additive model, **(C)** dominant model, and **(D)** recessive model].

**FIGURE 5:**
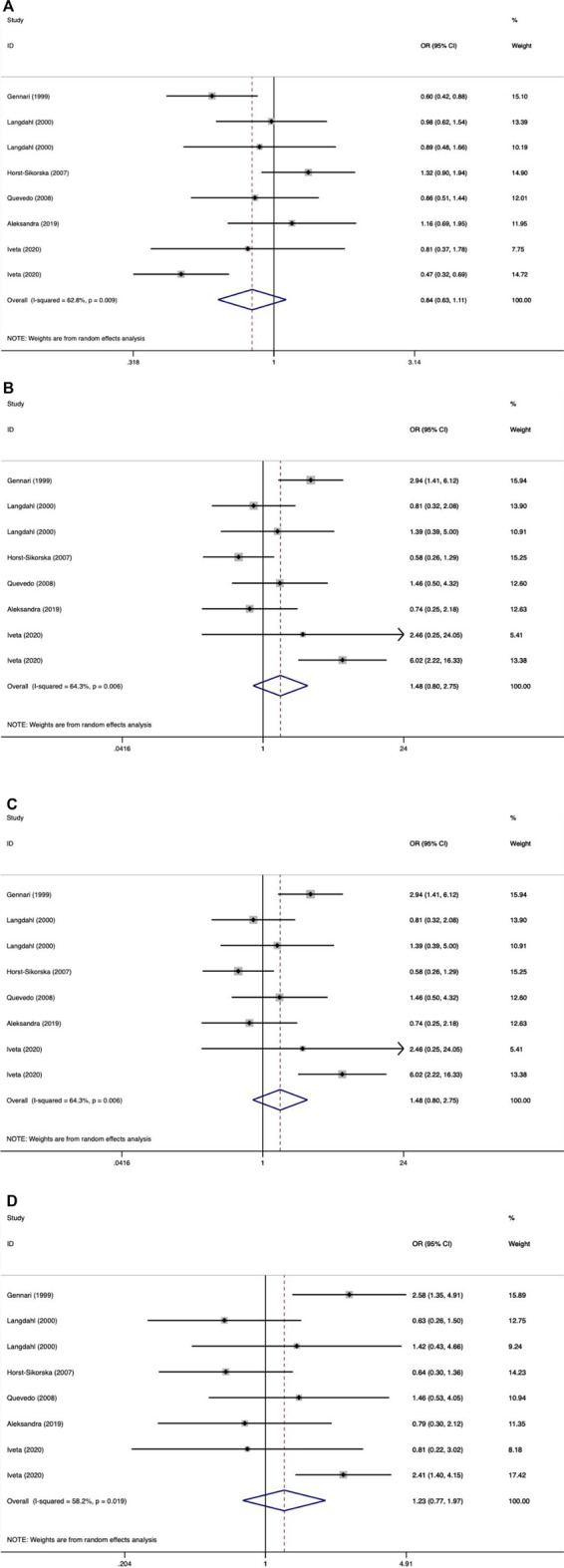
Forest plots of all selected studies on the association between VDR Fokl polymorphism and the risk of osteoporotic fracture in different races [**(A)** allele model, **(B)** additive model, **(C)** dominant model, and **(D)** recessive model].

**FIGURE 6:**
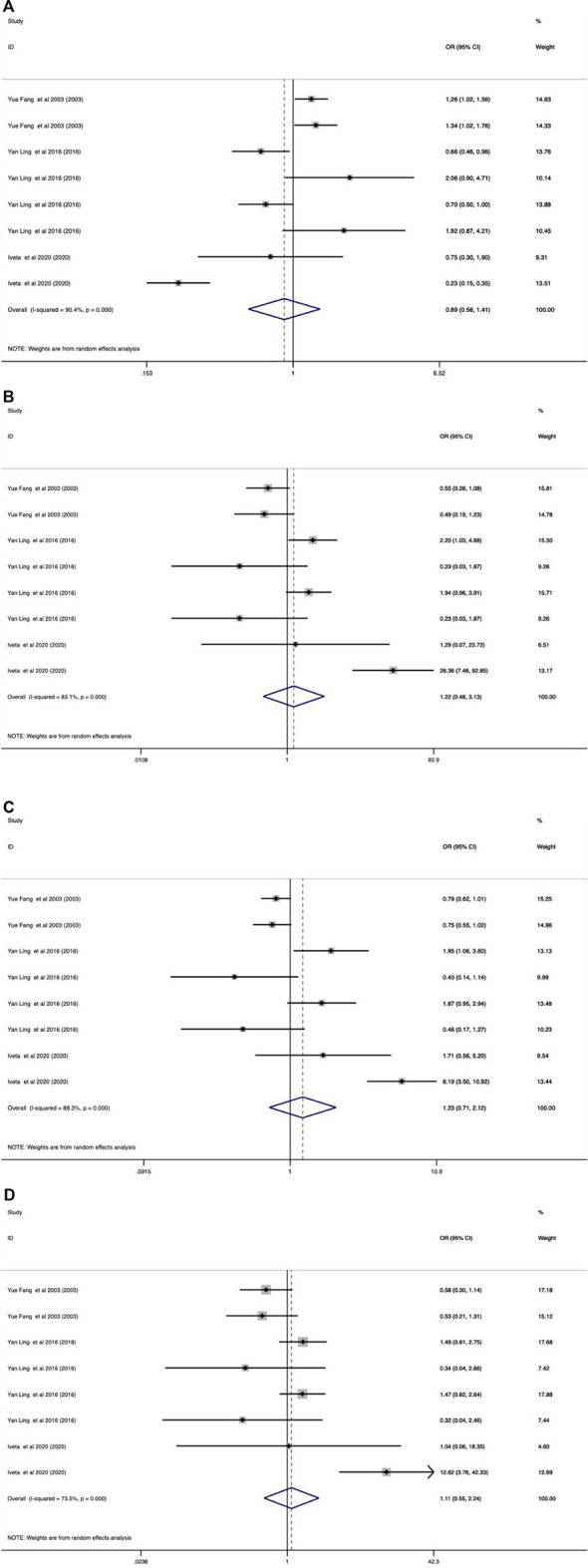
Forest plots of all selected studies on the association between VDR Cdx-2 polymorphism and the risk of osteoporotic fracture in different races [**(A)** allele model, **(B)** additive model, **(C)** dominant model, and **(D)** recessive model].

As shown in Tables 9–11 and Figures 4–6, there were no significant associations between the VDR TaqI, FokI, and Cdx2 polymorphisms and the risk of osteoporotic fractures.

**TABLE 9:** Pooled estimates of association of VDR TaqI polymorphism and the risk of osteoporotic fracture.

Genetic model	Variable	Test of association		Tests for heterogeneity		Egger’s test
		OR (95% CI)	*P*	*P*_*h*_	*I* ^*2*^ (%)	*P*_*E*_
T vs t	Overall	1,10 (0.83–1.47)	0.510	0.005	65.80	0.497
	Europe	1.09 (0.78–1.51)	0.623	0.002	70.70	
	Female	1.20 (0.81–1.78)	0.356	0.002	76.50	
	Male	0.78 (0.50–1.23)	0.284	0.538	0	
tt vs TT	Overall	0.82 (0.44–1.54)	0.544	0.004	66.40	0.549
	Europe	0.82 (0.40–1.68)	0.590	0.002	71.20	
	Female	0.70 (0.29–1.68)	0.422	0.001	77.80	
	Male	1.54 (0.51–4.67)	0.445	0.267	18.8	
Tt + tt vs TT	Overall	0.84 (0.62–1.14)	0.254	0.119	39.00	0.183
	Europe	0.87 (0.62–1.23)	0.439	0.085	46.00	
	Female	0.77 (0.53–1.13)	0.187	0.082	51.70	
	Male	1.36 (0.69–2.68)	0.379	0.463	0	
tt vs TT + Tt	Overall	0.91 (0.49–1.66)	0.749	0.001	70.20	0.276
	Europe	0.87 (0.43–1.75)	0.699	0.001	74.50	
	Female	0.79 (0.355–1.78)	0.568	0.001	78.20	
	Male	1.29 (0.21–8.00)	0.784	0.053	73.30	
TT + tt vs Tt	Overall	1.15 (0.87–1.50)	0.323	0.219	26.20	0.705
	Europe	1.10 (0.82–1.47)	0.537	0.194	30.70	
	Female	1.18 (0.92–1.52)	0.186	0.427	0.00	
	Male	0.97 (0.22–4.32)	0.968	0.024	80.30	

***VDR* TaqI**: allele model: T vs. t, additive model: tt vs. TT, dominant model: Tt + tt vs. TT, recessive model: tt vs. TT + Tt, over-dominant model: TT + tt vs. Tt.

**TABLE 10:** Pooled estimates of association of VDR FokI polymorphism and the risk of osteoporotic fracture.

Genetic model	Variable	Test of association		Tests for heterogeneity		Egger’s test
		OR (95% CI)	*P*	*P*_*h*_	*I* ^*2*^ (%)	*P*_*E*_
F vs f	Overall	0.84 (0.63–1.11)	0.210	0.009	62.80	0.609
	Europe	0.84 (0.61–1.15)	0.269	0.005	68.00	
	Female	0.79 (0.56–1.11)	0.178	0.005	70.20	
ff vs FF	Overall	1.48 (0.80–2.75)	0.212	0.006	64.30	0.949
	Europe	1.49 (0.73–3.03)	0.274	0.003	69.40	
	Female	1.68 (0.77–3.67)	0.188	0.003	71.90	
Ff + ff vs FF	Overall	1.27 (0.88–1.82)	0.196	0.071	46.30	0.199
	Europe	1.31 (0.86–2.00)	0.206	0.043	53.80	
	Female	1.43 (0.90–2.27)	0.134	0.036	58.00	
ff vs FF + Ff	Overall	1.23 (0.77–1.97)	0.377	0.019	58.20	0.122
	Europe	1.20 (0.71–2.03)	0.503	0.010	64.20	
	Female	1.28 (0.72–2.27)	0.400	0.009	67.40	
FF + ff vs Ff	Overall	0.97 (0.78–1.22)	0.821	0.684	0	0.237
	Europe	0.96 (0.76–1.22)	0.750	0.584	0.00	
	Female	0.96 (0.75–1.22)	0.719	0.463	0.00	

***VDR* FokI**: allele model: F vs. f, additive model: ff vs. FF, dominant model: Ff + ff vs. FF, recessive model: ff vs. FF + Ff, over-dominant model: FF + ff vs. Ff.

**TABLE 11:** Pooled estimates of association of VDR Cdx2 polymorphism and the risk of osteoporotic fracture.

Genetic model	Variable	Test of association		Tests for heterogeneity		Egger’s test
		OR (95% CI)	*P*	*P*_*h*_	*I* ^*2*^ (%)	*P*_*E*_
G vs A	Overall	0.89 (0.56–1.41)	0.628	<0.001	90.40	0.697
AA vs GG	Overall	1.22 (0.48–3.13)	0.679	<0.001	83.10	0.918
AG + AA vs GG	Overall	1.23 (0.71–2.12)	0.463	<0.001	88.30	0.434
AA vs GG + AG	Overall	1.11 (0.55–2.24)	0.764	<0.001	73.50	0.830
GG + AA vs AG	Overall	0.84 (0.57–1.23)	0.377	<0.001	76.60	0.385

**VDR Cdx2**: allele model: G vs. A, additive model: AA vs. GG, dominant model: AG + AA vs. GG, recessive model: AA vs. GG + AG, over-dominant model: GG + AA vs. AG.

Table 12 shows the results of articles that did not exclude HWD.

**TABLE 12:** Data related to the HWD article were not excluded.

Genetic model	Variable	Test of association	Tests for heterogeneity			Egger’s test
		OR (95% CI)	*P*	*P*_*h*_	*I* ^*2*^ (%)	*P*_*E*_
Pooled estimates of association of *VDR* BsmI polymorphism and the risk of osteoporotic fracture						
B vs b	Overall	0.96 (0.84–1.11)	0.60	<0.001	64.20	0.353
	Europe	0.92 (0.78–1.09)	0.322	<0.001	61.50	
	America	**1.17 (1.03–1.34)**	0.018	0.37	6.4	
	Female	0.95 (0.81–1.12)	0.564	<0.001	68.60	
	Male	1.09 (0.69–1.71)	0.709	0.183	41.1	
bb vs BB	Overall	1.07 (0.81–1.41)	0.635	<0.001	57.80	0.229
	Europe	1.20 (0.86–1.67)	0.289	0.002	56.20	
	America	**0.74 (0.58–0.94)**	0.012	0.480	0	
	Female	1.08 (0.79–1.47)	0.629	<0.001	62.80	
	Male	0.83 (0.38–1.82)	0.642	0.295	18.00	
Bb + bb vs BB	Overall	1.06 (0.87–1.30)	0.535	0.042	36.50	0.133
	Europe	1.18 (0.93–1.49)	0.171	0.100	32.10	
	America	0.83 (0.67–1.02)	0.079	0.455	0.00	
	Female	1.08 (0.86–1.36)	0.524	0.020	45.30	
	Male	0.91 (0.49–1.69)	0.756	0.823	0.00	
bb vs BB + Bb	Overall	1.03 (0.85–1.24)	0.774	<0.001	56.20	0.617
	Europe	1.08 (0.87–1.35)	0.471	0.007	52.00	
	America	**0.81 (0.66–0.99)**	0.040	0.01	66.80	
	Female	1.03 (0.84–1.27)	0.781	<0.001	60.90	
	Male	0.88 (0.40–1.95)	0.756	0.098	57.00	
BB + bb vs Bb	Overall	0.96 (0.86–1.06)	0.429	0.863	0.00	0.496
	Europe	0.99 (0.87–1.14)	0.935	0.893	0.00	
	America	0.93 (0.75–1.16)	0.527	0.315	15.60	
	Female	0.95 (0.85–1.96)	0.372	0.787	0.00	
	Male	0.95 (0.56–1.60)	0.846	0.305	15.80	
Pooled estimates of association of VDR ApaI polymorphism and the risk of osteoporotic fracture						
A vs a	Overall	0.86 (0.74–1.01)	0.072	0.094	38.30	0.220
	Europe	**0.83 (0.71–0.97)**	0.019	0.170	30.00	
	Female	0.84 (0.67–1.04)	0.104	0.031	56.90	
	Male	1.19 (0.75–1.91)	0.462	0.859	0	
aa vs AA	Overall	1.38 (0.99–1.93)	0.057	0.087	39.30	0.186
	Europe	**1.50 (1.09–2.07)**	0.012	0.168	30.20	
	Female	1.50 (0.97–2.32)	0.068	0.034	55.90	
	Male	0.57 (0.17–1.87)	0.353	0.604	0	
Aa + aa vs AA	Overall	1.21 (0.99–1.49)	0.063	0.482	0	0.947
	Europe	**1.26 (1.02–1.56)**	0.032	0.551	0	
	Female	1.27 (0.95–1.69)	0.103	0.192	30.90	
	Male	1.01 (0.47–2.14)	0.986	0.613	0	
aa vs AA + Aa	Overall	1.31 (1.00–1.73)	0.054	0.060	43.60	0.061
	Europe	**1.40 (1.07–1.83)**	0.015	0.105	38.00	
	Female	1.39 (0.99–1.94)	0.056	<0.040	54.60	
	Male	0.56 (0.19–1.58)	0.271	0.353	0	
AA + aa vs Aa	Overall	1.08 (0.90–1.30)	0.420	0.319	13.10	0.215
	Europe	1.08 (0.88–1.33)	0.443	0.248	21.10	
	Female	1.10 (0.88–1.36)	0.398	0.289	18.50	
	Male	0.70 (0.33–1.48)	0.349	0.277	15.20	
Pooled estimates of association of VDR TaqI polymorphism and the risk of osteoporotic fracture						
T vs t	Overall	1,15 (0.95–1.40)	0.159	0.011	56.40	0.466
	Europe	1.15 (0.93–1.42)	0.212	0.006	60.70	
	Female	1.22 (0.94–1.58)	0.138	0.004	68.70	
	Male	0.78 (0.50–1.23)	0.284	0.538	0	
tt vs TT	Overall	0.77 (0.49–1.21)	0.264	0.008	57.90	0.895
	Europe	0.77 (0.47–1.26)	0.297	0.005	62.20	
	Female	0.68 (0.38–1.20)	0.181	0.003	70.30	
	Male	1.54 (0.51–4.67)	0.445	0.267	18.8	
Tt + tt vs TT	Overall	0.82 (0.66–1.01)	0.061	0.187	27.00	0.336
	Europe	0.83 (0.67–1.05)	0.116	0.149	32.30	
	Female	0.80 (0.61–1.05)	0.101	0.103	43.20	
	Male	1.36 (0.69–2.68)	0.379	0.463	0	
tt vs TT + Tt	Overall	0.84 (0.54–1.32)	0.453	0.002	63.80	0.775
	Europe	0.82 (0.50–1.33)	0.421	0.001	67.10	
	Female	0.74 (0.43–1.26)	0.270	0.002	71.80	
	Male	1.29 (0.21–8.00)	0.784	0.053	73.30	
TT + tt vs Tt	Overall	1.09 (0.89–1.34)	0.387	0.217	23.80	0.743
	Europe	1.06 (0.86–1.31)	0.560	0.215	24.80	
	Female	1.05 (0.86–1.29)	0.615	0.375	6.90	
	Male	0.97 (0.22–4.32)	0.968	0.024	80.30	
Pooled estimates of association of VDR FokI polymorphism and the risk of osteoporotic fracture						
F vs f	Overall	0.83 (0.65–1.05)	0.121	0.016	57.50	0.573
	Europe	0.83 (0.63–1.08)	0.161	0.009	62.80	
	Female	0.79 (0.56–1.11)	0.178	0.005	70.20	
ff vs FF	Overall	1.53 (0.90–2.61)	0.116	0.011	59.90	0.996
	Europe	1.54 (0.85–2.81)	0.157	0.006	64.90	
	Female	1.68 (0.77–3.67)	0.188	0.003	71.90	
Ff + ff vs FF	Overall	122 (0.89–1.66)	0.220	0.100	40.10	0.153
	Europe	1.24 (0.87–1.78)	0.231	0.064	47.60	
	Female	1.43 (0.90–2.27)	0.134	0.036	58.00	
ff vs FF + Ff	Overall	1.34 (0.88–2.04)	0.167	0.020	56.10	0.086
	Europe	1.32 (0.83–2.10)	0.240	0.011	61.60	
	Female	1.28 (0.72–2.27)	0.400	0.009	67.40	
	Male	1.42 (0.43–4.66)	0.561			
FF + ff vs Ff	Overall	1.06 (0.86–1.30)	0.610	0.437	0	0.173
	Europe	1.05 (0.83–1.32)	0.711	0.337	12.00	
	Female	0.96 (0.75–1.22)	0.719	0.463	0.00	
Pooled estimates of association of VDR Cdx2 polymorphism and the risk of osteoporotic fracture						
G vs A	Overall	0.92 (0.63–1.11)	0.691	<0.001	89.50	0.599
AA vs GG	Overall	1.08 (0.46–2.53)	0.866	<0.001	82.60	0.903
AG + AA vs GG	Overall	1.17 (0.76–1.82)	0.477	<0.001	87.00	0.362
AA vs GG + AG	Overall	0.99 (0.52–1.89)	0.980	<0.001	73.30	0.762
GG + AA vs AG	Overall	0.88 (0.64–1.20)	0.403	<0.001	73.60	0.325

**VDR BsmI**: allele model: B vs. b, additive model: bb vs. BB, dominant model: Bb + bb vs. BB, recessive model: bb vs. BB+ Bb, over-dominant model: BB+ bb vs. Bb. **VDR ApaI:** allele model: A vs. a, additive model: aa vs. AA, dominant model: Aa + aa vs. AA, recessive model: aa vs. AA + Aa, over-dominant model: AA+ aa vs. Aa. **VDR TaqI**: allele model: T vs. t, additive model: tt vs. TT, dominant model: Tt + tt vs. TT, recessive model: tt vs. TT + Tt, over-dominant model: TT + tt vs. Tt. **VDR FokI**: allele model: F vs. f, additive model: ff vs. FF, dominant model: Ff + ff vs. FF, recessive model: ff vs. FF + Ff, over-dominant model: FF + ff vs. Ff. **VDR Cdx2**: allele model: G vs. A, additive model: AA vs. GG, dominant model: AG + AA vs. GG, recessive model: AA vs. GG + AG, over-dominant model: GG + AA vs. AG.

### Heterogeneity and Sensitivity Analyses

We observed heterogeneity in the overall and several subgroup analyses. Heterogeneity may be attributed to factors such as race, sex, and HWE. To explore the source of heterogeneity, a regression meta-analysis was used. However, no obvious source of heterogeneity was found by the results of regression meta-analyses. However, if it was taken into consideration that the previous exclusion of HWD-related articles leads to significant results in subgroup analysis, then it can be said that the source of heterogeneity might be HWD-related. Sensitivity analysis was estimated using three methods. First, a study was deleted every time to evaluate its robustness, and no change was observed in the research results. However, a significant change was observed in the obtained results once when low-quality and HWD studies were excluded. In previous studies, the VDR BsmI B allele increased the risk of osteoporotic fracture (OR 1.17, 95% CI 1.03–1.34), and the bb genotype reduced the risk of osteoporotic fracture in the United States (additive model: OR 0.74, 95% CI 0.88–0.94; allelic model: OR 0.81, 95% CI 0.66–0.99), but after excluding low-quality and HWD studies, the results showed no significant association between VDR BsmI gene polymorphism and fracture risk in the American population (allelic model: OR 1.18, 95% CI 0.82–1.70; additive model: OR 0.73, 95% CI 0.38–1.40; recessive model: OR 0.91, 95% CI 0.59–1.42). In addition, an increased risk of osteoporosis fracture was found in individuals with the AA genotype only in the European population (allele model: OR 0.83, 95% CI 0.70–0.98; additive model: OR 1.52, 95% CI 1.07–2.16; dominant model: OR 1.26, 95% CI 1.01–1.57; recessive model: OR 1.42, 95% CI 1.06–1.90), which was also different from previous studies (allelic model: OR 0.83, 95% CI 0.71–0.97; additive model: OR 1.50, 95% CI 1.09–2.07; dominant model: OR 1.26, 95% CI 1.02–1.56; recessive model: OR 1.40, 95% CI 1.07–1.83). In addition, when the studies were limited to only high quality, HWE, and matching, the corresponding total OR value was not significantly changed. The sensitivity analysis results are presented in Table 13.

**TABLE 13:** Pooled estimates of association of VDR BsmI, ApaI, TaqI, and FokI polymorphisms and the risk of osteoporotic fracture, excluding low-quality and HWD studies.

Genetic model	Test of association		Tests for heterogeneity	
	OR (95% CI)	*P*	*P*_*h*_	*I* ^*2*^ (%)
VDR BsmI				
B vs b	0.93 (0.79–1.08)	0.339	0.000	61.60
bb VS BB	1.15 (0.84–1.58)	0.370	0.001	56.70
Bb + bb VS BB	1.13 (0.90–1.43)	0.298	0.042	38.50
bb VS BB + Bb	1.09 (0.89–1.32)	0.415	0.006	50.20
BB + bb VS Bb	1.01 (0.79–1.15)	0.868	0.872	0
*VDR* ApaI				
A vs a	0.86 (0.73–1.03)	0.100	0.063	44.3
aa VS AA	1.39 (0.96–1.99)	0.079	0.059	45.1
Aa + aa VS AA	1.21 (0.97–1.50)	0.086	0.391	5.5
aa VS AA + Aa	1.33 (0.99–1.78)	0.063	0.044	48.1
AA + aa VS Aa	1.09 (0.90–1.33)	0.383	0.269	18.9
*VDR* TaqI				
T vs t	1.09 (0.78–1.51)	0.624	0.002	70.7
tt VS TT	0.83 (0.40–1.71)	0.611	0.002	71.2
Tt + tt VS TT	0.86 (0.61–1.22)	0.390	0.076	47.6
tt VS TT + Tt	0.90 (0.45–1.82)	0.770	0.001	74.4
TT + tt VS Tt	1.13 (0.83–1.54)	0.441	0.150	36.4
VDR FokI				
F vs f	0.90 (0.67–1.21)	0.495	0.076	52.8
ff VS FF	1.23 (0.64–2.38)	0.532	0.045	58.9
Ff + ff VS FF	1.12 (0.83–1.49)	0.464	0.368	6.8
ff VS FF + Ff	1.17 (0.63–2.19)	0.621	0.033	61.8
FF + ff VS Ff	0.98 (0.74–1.29)	0.868	0.535	0

### Publication Bias

**FIGURE 7:**
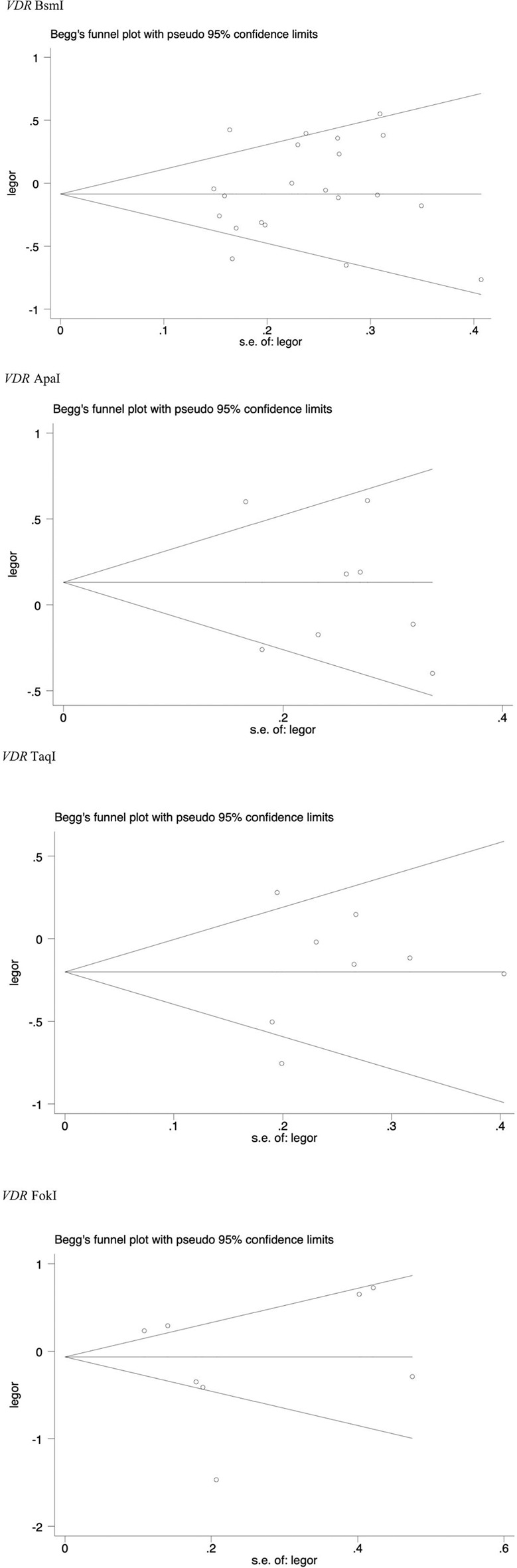
Begg’s funnel plot to access publication bias.

Publication bias was evaluated using Begg’s funnel plot and Egger’s test. The shape of the funnel plot shows that there was no obvious funnel asymmetry in the entire population (Figure 7). Egger’s test also showed no evidence of significant publication bias (*p* > 0.05 in all genetic models), as displayed in Tables 7–11.

### Credibility of the Identified Genetic Associations

We determined that significant associations meeting the following statistical criteria were classified as “positive results” (Montazeri et al., 2019): 1) the P value of the Z-test <0.05 in at least two gene models; 2) at the P value level of 0.05, the FPRP was <0.2; 3) statistical power >0.8; and 4) I^2^ < 50%. Results were considered as “less credible results” with a lower threshold when the following conditions were met: 1) *p* < 0.05 in at least one of the genetic models; 2) the statistical power was between 50 and 79%, FPRP >0.2, or I^2^ > 50%. After confidence evaluation, it was determined that the statistically significant associations in this meta-analysis were “unreliable.” The detailed confidence evaluation results are presented in Table 14.

**TABLE 14:** False-positive report probability values for the statistically significant associations in the current meta-analysis.

Variables	OR (95% CI)	*I* ^*2*^ (%)	Statistical power		Prior probability of 0.001	
			0R = 1.2	OR = 1.5	0R = 1.2	OR = 1.5
Europe						
A vs a	0.83 (0.71–0.97)	30.00	0.480	0.997	0.976	0.950
aa vs AA	1.50 (1.09–2.07)	30.20	0.087	0.500	0.994	0.965
Aa + aa vs AA	1.26 (1.02–1.56)	0	0.327	0.945	0.990	0.973
aa vs AA + Aa	1.40 (1.07–1.83)	38.00	0.130	0.693	0.991	0.952

## Discussion

Osteoporosis is characterized by decreased bone density and increased bone fragility, which leads to increased fracture risk (Recker, 2005). Genes play an important role in the development of osteoporotic fractures, and the VDR gene has been extensively studied as a candidate gene that plays a key role in regulating bone resorption and metabolism (Jin and Ralston, 2001; Recker and Deng, 2002), and influencing bone mass (Kim et al., 2007). Therefore, it is important to study the relationship between VDR polymorphisms and osteoporotic fracture risk. Many previous studies have attempted to clarify the relationship between the polymorphisms of VDR and the risk of osteoporotic fracture. Unfortunately, there is no reliable evidence to show whether there is a relationship between them, which may be due to different reasons, including small sample size, race, and regional differences. Therefore, a meta-analysis is a valid alternative.

This meta-analysis included 23 studies, among which 18 explored the relationship between the VDR polymorphism BsmI and osteoporosis fracture risk, eight studies reported VDR ApaI polymorphism, nine studies reported VDR TaqI polymorphism, seven studies reported VDR FokI polymorphism, and three studies were related to VDR Cdx2 polymorphism. In addition, five genetic models were compared. Overall, the VDR BsmI polymorphism had no significant effect on the risk of osteoporotic fractures. However, in subgroup analysis, there was a significant correlation between the two. Moreover, the VDR ApaI polymorphism also did not significantly affect the risk of osteoporotic fracture. According to racial stratification, it was found that the genotype aa increased the risk of osteoporotic fracture in European countries compared with the AA genotype. However, no meaningful results were found regarding the relationship between the VDR polymorphisms (TaqI, VDR FokI, and Cdx2) and osteoporotic fracture. Moreover, when the low-quality and HWD research were excluded, and when the combined analysis involved only high-quality, HWE, and matching research, no significant correlation was observed. Furthermore, the current meta-analysis was carried out by applying multiple subgroups and different genetic models at the expense of multiple comparisons; in this case, the aggregated P value must be adjusted (Attia et al., 2003). The Venice standard, statistical ability, and I^2^ value are important standards (Langdahl et al., 2000). Therefore, the FPRP and Venice standards were used to evaluate positive results. After the credibility evaluation, it was determined that “positive results are not credible,” which are statistically significant in the current meta-analysis. After the regression meta-analysis, no source of obvious heterogeneity was identified. In addition, no obvious asymmetry was found in the study of VDR BsmI, ApaI, TaqI, and FokI polymorphisms using Begg’s funnel plot and Egger’s test. However, owing to the limited number of studies, Begg’s funnel plot was not used to explore publication deviation in VDR Cdx2 research. Finally, Egger’s test showed that there was no clear statistical evidence to show publication bias.

Four meta-analyses analyzed the association between VDR polymorphisms and risk of osteoporotic fracture. Fang et al. (Shen et al., 2014), Shen et al. (Moher et al., 2009), and Gao et al. (Aerssens et al., 2000) discussed the association between the VDR BsmI polymorphism and the risk of osteoporotic fracture, and their results showed that there was no significant association between VDR BsmI polymorphism and the risk of osteoporotic fracture. However, Ji et al. (Gao et al., 2015) examined 17 studies on the relationship of VDR BsmI polymorphism with osteoporotic fracture risk, including 2,112 osteoporotic fracture cases and 4,521 controls, and indicated that there was a statistically significant association between the VDR BsmI polymorphism and osteoporotic fracture risk. In addition, Fang et al. (Shen et al., 2014) and Shen et al. (Moher et al., 2009) examined four and five VDR TaqI studies, respectively, all of which considered that the VDR TaqI polymorphism was not significantly associated with osteoporotic fracture risk. Four studies on VDR ApaI and four studies on VDR FokI analyzed by Shen et al. (Moher et al., 2009) did not find that the VDR ApaI and FokI polymorphisms increased the risk of osteoporotic fracture. In addition, some shortcomings were found when published meta-analyses were carefully checked. First, there was no quality evaluation for the included studies in the two meta-analyses (Shen et al., 2014; Gao et al., 2015), and low-quality studies might have been included, which led to a deviation in the results. Second, the genotype distribution in the control group was not detected by the HWE (Moher et al., 2009; Shen et al., 2014; Gao et al., 2015). The HWE is necessary for a sound genetic association study. If the control group does not meet the requirements of the HWE, there may be selection bias or genotype errors, thus making the results unreliable. Third, the statistical power was not calculated in some previous meta-analyses (Moher et al., 2009; Shen et al., 2014; Gao et al., 2015). At the same time, the statistically significant false-positive report probability was not evaluated in all previously published meta-analyses. Therefore, the meta-analysis results may not be credible. Finally, none of the abovementioned studies discussed the relationship between the VDR Cdx2 polymorphism and osteoporotic fracture.

This meta-analysis had the following advantages: 1) evaluating the quality of the included research; 2) the control group underwent the HWE test; 3) applying the FPRP and Venice criteria to evaluate the correlations that were found to be significant in the current meta-analysis; 4) compared with the previous meta-analysis, the sample size has been significantly expanded; and 5) exploring the sources of heterogeneity based on regression meta-regression analysis. However, there are still some limitations to this study. First, the confounding factors closely related to the outcome were not controlled, such as smoking, drinking, and variable research designs. Second, there are relatively few studies on Americans and Asians in several subgroup analyses, and not enough statistical power to explore the real connection. Moreover, owing to the limited number of studies, a subgroup analysis was not carried out in the summary analysis of the VDR Cdx2 polymorphism and osteoporotic fracture risk. Finally, it was found that the research quality of VDR Cdx2 is low, and hence, the results may not be credible. Future research with large sample sizes and large enough subgroups will help verify our findings.

This meta-analysis strongly indicates that there is no significant association between the polymorphisms of VDR BsmI, ApaI, TaqI, FokI, and Cdx2 and the risk of osteoporotic fracture. The increased risk of osteoporotic fracture elucidated in previous studies is most likely due to false-positive results.

## Data Availability

The original contributions presented in the study are included in the article/Supplementary Material; further inquiries can be directed to the corresponding authors.

## Abbreviations

BMD, bone mineral density; 95% CI, 95% confidence interval; Fn, femoral neck; FPRP, false-positive report probabilities; HWD, Hardy–Weinberg disequilibrium; HWE, Hardy–Weinberg equilibrium (ideally, the frequency of alleles is constant in heredity; that is, gene balance is maintained); LS, lumbar spine; OR, odds ratio; VDR, vitamin D receptor; PRISMA, preferred reporting items for systematic review and meta-analyses; SNP, single-nucleotide polymorphism.

## References

[B1] Aerssens J. D(equeker J. Peeters J. Breemans S. Broos P. Boonen S. (2000). Polymorphisms of the VDR, ER and COLIA1 Genes and Osteoporotic Hip Fracture in Elderly Postmenopausal Women. Osteoporos. Int. 11, 583–591. 10.1007/s001980070079 11069192

[B2] Alvarez-Hernández D. Naves M. Díaz-López J. B. Gómez C. Santamaría I. Cannata-Andía J. B. (2003). Influence of Polymorphisms in VDR and COLIA1 Genes on the Risk of Osteoporotic Fractures in Aged Men. Kidney International. Suppl. 63 (85), S14–S18. 10.1046/j.1523-1755.63.s85.5.x 12753258

[B3] Attia J. Thakkinstian A. D'Este C. (2003). Meta-analyses of Molecular Association Studies: Methodologic Lessons for Genetic Epidemiology. J. Clin. Epidemiol. 56 (4), 297–303. 10.1016/s0895-4356(03)00011-8 12767405

[B4] Binici D. N. Gunes N. (2010). Risk Factors Leading to Reduced Bone mineral Density in Hemodialysis Patients with Metabolic Syndrome. Ren. Fail. 32 (4), 469–474. 10.3109/08860221003675260 20446786

[B5] Iveta B. Jarmila B. Soňa M. Ján K. Zlatica T. Ivan B. (2020). Association between Vitamin D Receptor Gene Polymorphisms (Fok I, Cdx-2) and Bone mineral Density in Slovak Postmenopausal Women. Anthropologischer Anzeiger 77, 195–203. 10.1127/anthranz/2020/1048 32236287

[B6] Cummings S. R. Melton L. J. (2002). Epidemiology and Outcomes of Osteoporotic Fractures. The Lancet 359, 1761–1767. 10.1016/s0140-6736(02)08657-9 12049882

[B7] Efesoy A. Yilmaz Ö. Erden G. Güçtekin A. Bodur H. Yildirimkaya M (2003). Relationship of the Vitamin D Receptor and Collagen Iα1 Gene Polymorphisms with Low Bone Mineral Density and Vertebral Fractures in Postmenopausal Turkish Women. Turk J. Rheumatol. 26, 295–302. 10.5606/tjr.2011.047

[B8] Fang Y. Rivadeneira F. van Meurs J. B. J. Pols H. A. P. Ioannidis J. P. A. Uitterlinden A. G. (2006). Vitamin D Receptor Gene BsmI and TaqI Polymorphisms and Fracture Risk: a Meta-Analysis. Bone 39 (4), 938–945. 10.1016/j.bone.2006.04.016 16769262

[B9] Fang Y. Van Meurs J. B. Bergink A. P. Hofman A. Van Duijn C. M. Van Leeuwen J. P. (2003). Cdx-2 Polymorphism in the Promoter Region of the Human Vitamin D Receptor Gene Determines Susceptibility to Fracture in the Elderly. J. Bone Miner. Res. 18 (9), 1632–1641. 10.1359/jbmr.2003.18.9.1632 12968672

[B10] Feskanich D. Hunter D. J. Willett W. C. Hankinson S. E. Hollis B. W. Hough H. L. (1998). Vitamin D Receptor Genotype and the Risk of Bone Fractures in Women. Epidemiology 9, 535–539. 10.1097/00001648-199809000-00011 9730033

[B11] Gao J. Wang L. Zhu J. (2015). Influence of BsmI Polymorphism in Vitamin D Receptor Gene on the Risk of Fracture in Caucasian Populations: a Meta Analysis. Int. J. Clin. Exp. Med. 8 (1), 589–597. 25785033 PMC4358488

[B12] Garnero P. Munoz F. Borel O. Sornay-Rendu E. Delmas P. D. (2005). Vitamin D Receptor Gene Polymorphisms Are Associated with the Risk of Fractures in Postmenopausal Women, Independently of Bone mineral Density. J. Clin. Endocrinol. Metab. 90 (8), 4829–4835. 10.1210/jc.2005-0364 15886235

[B13] Gennari L. Becherini L. Mansani R. Masi L. Falchetti A. Morelli A. (1999). FokI Polymorphism at Translation Initiation Site of the Vitamin D Receptor Gene Predicts Bone Mineral Density and Vertebral Fractures in Postmenopausal Italian Women. J. Bone Mineral Res. 14, 1379–1386. 10.1359/jbmr.1999.14.8.1379 10457270

[B14] Gómez C. Naves M. L. Barrios Y. Díaz J. B. Fernández J. L. Salido E. (1999). Vitamin D Receptor Gene Polymorphisms, Bone Mass, Bone Loss and Prevalence of Vertebral Fracture: Differences in Postmenopausal Women and Men. Osteoporos. Int. 10 (3), 175–182. 10.1007/s001980050213 10525708

[B15] Horst-Sikorska W. Wawrzyniak A. Celczyńska-Bajew L. Marcinkowska M. Dabrowski S. Kalak R. (2005). Polimorfizm Genu VDR-Efektywny Marker Molekularny Ryzyka Osteoporotycznych Złamań Kości W Grupie Kobiet Po Menopauzie Pochodzących Z Rejonu. Endokrynol Pol. 56, 233–239. 16350715

[B16] Horst-Sikorska W. Dytfeld J. Wawrzyniak A. Marcinkowska M Michalak M Franek E (2013). Vitamin D Receptor Gene Polymorphisms, Bone mineral Density and Fractures in Postmenopausal Women with Osteoporosis. Mol. Biol. Rep. 40 (1), 383–390. 10.1007/s11033-012-2072-3 23070909 PMC3518805

[B17] Horst-Sikorska W. Kalak R. Wawrzyniak A. Marcinkowska M. Celczynska-Bajew L. Slomski R. (2007). Association Analysis of the Polymorphisms of the VDR Gene with Bone mineral Density and the Occurrence of Fractures. J. Bone Miner. Metab. 25, 310–319. 10.1007/s00774-007-0769-5 17704996

[B18] Houston L. A. Grant S. F. A. Reid D. M. Ralston S. H. (1996). Vitamin D Receptor Polymorphism, Bone mineral Density, and Osteoporotic Vertebral Fracture: Studies in a UK Population. Bone 18, 249–252. 10.1016/8756-3282(95)00483-1 8703580

[B19] Iván Q. L. Milka M. B. Marcelo C. N. Nancy R. F. (2008). Polimorfismos del gen del receptor de vitamina D y riesgo de fractura de cadera en la mujer adulta mayor de la Región del Bío Bío. Revista Médica De Chile 136 (4), 475–481. 10.4067/s0034-98872008000400008 18769790

[B20] Aleksandra J. -P. Jowita H. -Z. Katarzyna K. Łukasz G. Agnieszka Z. Marek B. (2019). Association of Vitamin D Receptor Polymorphisms with Activity of Acromegaly, Vitamin D Status and Risk of Osteoporotic Fractures in Acromegaly Patients. Front. Endocrinol. (Lausanne) 10, 643. 10.3389/fendo.2019.00643 31616375 PMC6768940

[B21] Ji G.-R. Yao M. Sun C.-Y. Li Z.-H. Han Z. BsmI Taq. I. (2010). BsmI, TaqI, ApaI and FokI Polymorphisms in the Vitamin D Receptor (VDR) Gene and Risk of Fracture in Caucasians: A Meta-Analysis. Bone 47 (3), 681–686. 10.1016/j.bone.2010.06.024 20601302

[B22] Jin H. Ralston S. H. (2001). Genetics of Osteoporosis. Rev. Endocr. Metab. Disord. 2 (1), 13–21. 11704976 10.1023/a:1010098706338

[B23] Karpiński M. Galicka A. Milewski R. Popko J. Badmaev V. Stohs S. J. (2017). Association between Vitamin D Receptor Polymorphism and Serum Vitamin D Levels in Children with Low-Energy Fractures. J. Am. Coll. Nutr. 36 (1), 64–71. 10.1080/07315724.2016.1218803 28067591

[B24] Kaufman J.-M. Ostertag A. Saint-Pierre A. Cohen-Solal M. Boland A. Van Pottelbergh I. (2008). Genome-wide Linkage Screen of Bone mineral Density (BMD) in European Pedigrees Ascertained through a Male Relative with Low BMD Values: Evidence for Quantitative Trait Loci on 17q21-23, 11q12-13, 13q12-14, and 22q11. J. Clin. Endocrinol. Metab. 93 (10), 3755–3762. 10.1210/jc.2008-0678 18664539

[B25] Kim M.-s. Fujiki R. Murayama A. Kitagawa H. Yamaoka K. Yamamoto Y. (2007). 1α,25(OH)2D3-Induced Transrepression by Vitamin D Receptor through E-box-type Elements in the Human Parathyroid Hormone Gene Promoter. Mol. Endocrinol. 21, 334–342. 10.1210/me.2006-0231 17095575

[B26] Langdahl B. L. Gravholt C. H. Brixen K. Eriksen E. F. (2000). Polymorphisms in the Vitamin D Receptor Gene and Bone Mass, Bone Turnover and Osteoporotic Fractures. Eur. J. Clin. Invest. 30, 608–617. 10.1046/j.1365-2362.2000.00686.x 10886301

[B27] Ling Y. Lin H. Aleteng Q. Ma H. Pan B. Gao J. (2016). Cdx-2 Polymorphism in Vitamin D Receptor Gene Was Associated with Serum 25-hydroxyvitamin D Levels, Bone mineral Density and Fracture in Middle-Aged and Elderly Chinese Women. Mol. Cell. Endocrinol. 427, 155–161. 10.1016/j.mce.2016.03.014 26970179

[B28] Moher D. Liberati A. Tetzlaff J. Altman D. G. (2009). Preferred Reporting Items for Systematic Reviews and Meta-Analyses: the PRISMA Statement. J. Clin. Epidemiol. 62 (10), 1006–1012. 10.1016/j.jclinepi.2009.06.005 19631508

[B29] Montazeri Z. Li X. Nyiraneza C. Ma X. Timofeeva M. Svinti V. (2019). Systematic Meta-Analyses, Field Synopsis and Global Assessment of the Evidence of Genetic Association Studies in Colorectal Cancer. Gut 69, 1460–1471. 10.1136/gutjnl-2019-319313 31818908 PMC7398467

[B31] Ng M. Y. Sham P. C. Paterson A. D. Chan V. Kung A. W. (2006). Effect of Environmental Factors and Gender on the Heritability of Bone mineral Density and Bone Size. Ann. Hum. Genet. 70 (Pt 4), 428–438. 10.1111/j.1469-1809.2005.00242.x 16759177

[B32] Nguyen T. V. Esteban L. M. White C. P. Grant S. F. Center J. R. Gardiner E. M. (2005). Contribution of the Collagen I Alpha1 and Vitamin D Receptor Genes to the Risk of Hip Fracture in Elderly Women. J. Clin. Endocrinol. Metab. 90 (12), 6575–6579. 10.1210/jc.2005-1153 16159929

[B44] Quevedo L. I. Martínez B. M. Castillo N. M. Rivera F N. (2008). Polimorfismos del gen del receptor de vitamina D y riesgo de fractura de cadera en la mujer adulta mayor de la Región del Bío Bío (Vitamin D Receptor Gene Polymorphisms and Risk of Hip Fracture in Chilean Elderly Women]. Rev. Med. Chil. 136 (4), 475–81. [Epub ahead of print]. 18769790

[B33] Ramalho A. C. Lazaretti-Castro M. Hauache O. Kasamatsu T. Brandão C. Reis A. F. (1998). Fractures of the Proximal Femur: Correlation with Vitamin D Receptor Gene Polymorphism. Braz. J. Med. Biol. Res. 31, 921–927. 10.1590/s0100-879x1998000700006 9698755

[B34] Recker R. R. (2005). Summary--Novel Therapies for Osteoporosis. J. Musculoskelet. Neuronal Interact 5, 358–359. 16340138

[B35] Recker R. R. Deng H.-W. (2002). Role of Genetics in Osteoporosis. Endo. 17, 55–66. 10.1385/endo:17:1:55 12014706

[B36] Saccone D. Asani F. Bornman L. (2015). Regulation of the Vitamin D Receptor Gene by Environment, Genetics and Epigenetics. Gene 561 (2), 171–180. 10.1016/j.gene.2015.02.024 25682935

[B37] Seuter S. Neme A. Carlberg C. (2016). Epigenome-wide Effects of Vitamin D and Their Impact on the Transcriptome of Human Monocytes Involve CTCF. Nucleic Acids Res. 44 (9), 4090–4104. 10.1093/nar/gkv1519 26715761 PMC4872072

[B38] Shen H. Xie J. Lu H. (2014). Vitamin D Receptor Gene and Risk of Fracture in Postmenopausal Women: a Meta-Analysis. Climacteric 17 (4), 319–324. 10.3109/13697137.2013.856401 24156276

[B39] Uitterlinden A. G. Weel A. Burger H. Fang Y. Van Duijn C. M. Hofman A. (2001). Interaction between the Vitamin D Receptor Gene and Collagen Type I1 Gene in Susceptibility for Fracture. J. Bone Mineral Res. 16, 379–385. 10.1359/jbmr.2001.16.2.379 11204438

[B40] Uitterlinden A. G. Fang Y. van Meurs J. B. J. Pols H. A. P. van Leeuwen J. P. T. M. (2004). Genetics and Biology of Vitamin D Receptor Polymorphisms. Gene 338 (2), 143–156. 10.1016/j.gene.2004.05.014 15315818

[B41] Uzzan B. Cohen R. Nicolas P. Cucherat M. Perret G. Y. (2007). Effects of Statins on Bone mineral Density: a Meta-Analysis of Clinical Studies. Bone 40, 1581–1587. 10.1016/j.bone.2007.02.019 17409043

[B42] Välimäki S. Tähtelä R. Kainulainen K. Laitinen K. Löyttyniemi E. Sulkava R. (2001). Relation of Collagen Type I Alpha 1 (COLIA 1) and Vitamin D Receptor Genotypes to Bone Mass, Turnover, and Fractures in Early Postmenopausal Women and to Hip Fractures in Elderly People. Eur. J. Intern. Med. 12 (1), 48–56. 10.1016/s0953-6205(00)00137-0 11173011

[B43] Wengreen H. Cutler D. R. Munger R. Willing M. (2006). Vitamin D Receptor Genotype and Risk of Osteoporotic Hip Fracture in Elderly Women of Utah: an Effect Modified by Parity. Osteoporos. Int. 17, 1146–1153. 10.1007/s00198-006-0100-7 16758135

