# Supplementary material for: Evaluation of Association Studies and an Updated Meta-Analysis of VDR Polymorphisms in Osteoporotic Fracture Risk
Source: Front Genet. 2022 Jan 7;12:791368. doi: 10.3389/fgene.2021.791368 (PMC8782145; doi:10.3389/fgene.2021.791368)
Supplement: Supplementary file 1 [file Table1.docx]

| **Supplementary Table 1 Scale for quality assessment of molecular association studies** | |
| --- | --- |
| Criterion | Score |
| Source of case | |
| Selected from population | 2 |
| Selected from hospital | 1 |
| Not described | 0 |
| Source of control | |
| Population-based | 3 |
| Blood donors or volunteers | 2 |
| Hospital-based | 1 |
| Not described | 0 |
| Ascertainment of osteoporotic fracture | |
| WHO | **2** |
| Diagnosis of osteoporotic fracture by patient medical record | 1 |
| Not described | 0 |
| Ascertainment of control | |
| Controls were tested to screen out | 2 |
| Controls were subjects who did not report osteoporosis, no objective testing | 1 |
| Not described | 0 |
| Matching | |
| Controls matched with cases by age and sex | 2 |
| Controls matched with cases only by age or sex | 1 |
| Not matched or not described | 0 |
| Genotyping examination | |
| Genotyping done blindly and quality control | 2 |
| Only genotyping done blindly or quality control | 1 |
| Unblinded and without quality control | 0 |
| Specimens used for determining genotypes | |
| Blood cells or normal tissues | 1 |
| Tumor tissues or exfoliated cells of tissue | 0 |
| HWE | |
| HWE in the control group | 1 |
| Hardy-Weinberg disequilibrium in the control group | 0 |
| Association assessment | |
| Assess association between genotypes and osteoporotic fracture with appropriate statistics and adjustment for confounders | 2 |
| Assess association between genotypes and osteoporotic fracture with appropriate statistics without adjustment for confounders | 1 |
| Inappropriate statistics used | 0 |
| Total sample size |  |
| >500 | 3 |
| 200-500 | 2 |
| <200 | 1 |
| HWE: Hardy-Weinberg equilibrium |  |
